# Molecular Analysis of the *Retinoic Acid Induced 1* Gene (*RAI1*) in Patients with Suspected Smith-Magenis Syndrome without the 17p11.2 Deletion

**DOI:** 10.1371/journal.pone.0022861

**Published:** 2011-08-08

**Authors:** Thierry Vilboux, Carla Ciccone, Jan K. Blancato, Gerald F. Cox, Charu Deshpande, Wendy J. Introne, William A. Gahl, Ann C. M. Smith, Marjan Huizing

**Affiliations:** 1 Medical Genetics Branch, National Human Genome Research Institute, National Institutes of Health, Bethesda, Maryland, United States of America; 2 Department of Oncology, Georgetown University Medical Center, Washington, D.C., United States of America; 3 Division of Genetics, Department of Pediatrics, Harvard Medical School, Children's Hospital Boston, Boston, Massachusetts, United States of America; 4 Department of Genetics, Guy's Hospital, London, United Kingdom; 5 Office of the Clinical Director, National Human Genome Research Institute, National Institutes of Health, Bethesda, Maryland, United States of America; 6 Genzyme Corporation, Cambridge, Massachusetts, United States of America; Ohio State University Medical Center, United States of America

## Abstract

Smith-Magenis syndrome (SMS) is a complex neurobehavioral disorder characterized by multiple congenital anomalies. The syndrome is primarily ascribed to a ∼3.7 Mb *de novo* deletion on chromosome 17p11.2. Haploinsufficiency of multiple genes likely underlies the complex clinical phenotype. *RAI1* (*Retinoic Acid Induced 1*) is recognized as a major gene involved in the SMS phenotype. Extensive genetic and clinical analyses of 36 patients with SMS-like features, but without the 17p11.2 microdeletion, yielded 10 patients with *RAI1* variants, including 4 with *de novo* deleterious mutations, and 6 with novel missense variants, 5 of which were *familial*. Haplotype analysis showed two major *RAI1* haplotypes in our primarily Caucasian cohort; the novel *RAI1* variants did not occur in a preferred haplotype. RNA analysis revealed that *RAI1* mRNA expression was significantly decreased in cells of patients with the common 17p11.2 deletion, as well as in those with *de novo RAI1* variants. Expression levels varied in patients with familial *RAI1* variants and in non-17p11.2 deleted patients without identified *RAI1* defects. No correlation between SNP haplotype and *RAI1* expression was found. Two clinical features, ocular abnormalities and polyembolokoilomania (object insertion), were significantly correlated with decreased *RAI1* expression. While not significantly correlated, the presence of hearing loss, seizures, hoarse voice, childhood onset of obesity and specific behavioral aspects and the absence of immunologic abnormalities and cardiovascular or renal structural anomalies, appeared to be specific for the *de novo RAI1* subgroup. Recognition of the combination of these features will assist in referral for *RAI1* analysis of patients with SMS-like features without detectable microdeletion of 17p11.2. Moreover, *RAI1* expression emerged as a genetic target for development of therapeutic interventions for SMS.

## Introduction

Smith-Magenis syndrome (SMS; OMIM 182290) is a complex neurobehavioral syndrome characterized by multiple congenital anomalies and behavior problems, including craniofacial and skeletal abnormalities, variable intellectual disability, self-injurious and attention-seeking behaviors, speech and motor delay, and sleep disturbance [1], [2], [3], [4], [5]. The estimated prevalence of SMS in the general population is ∼1∶15000–25000, but it is likely underdiagnosed [6]. The syndrome is caused primarily by *de novo* interstitial deletions of chromosome 17p11.2, which can range from 1.5 to 9 megabases (Mb) in size, detectable by cytogenetic G-banding and/or by fluorescence *in situ* hybridization (FISH) analysis. The most common ∼3.7 Mb deletion occurs in approximately 75% of the patients [3], [4], [5], [7], [8].

Several genes have been mapped to the 17p11.2 SMS critical region, and the exact functions of many of these genes remain unknown [5], [9], [10]. Haploinsufficiency for several genes is likely to account for the SMS phenotype, but haploinsufficiency for the retinoic acid induced 1 gene (*RAI1*), located within the minimal critical SMS deletion region, is considered to play a major role in SMS. This is supported by the identification of heterozygous point mutations in *RAI1* in SMS patients without detectable 17p11.2 deletions. Such individuals share most, but not all, characteristics of the SMS phenotype [11], [12], [13], [14], but their levels of *RAI1* mRNA transcription and RAI1 protein translation have not been assessed.

The *RAI1* gene (OMIM 607642; GenBank NM_030665) consists of 6 exons, of which exons 3 through 6 encode a 1,906 amino acid RAI1 protein [15]. An *RAI1* mRNA transcript of approximately 8 kb is expressed in all adult and fetal tissues examined [16], with heart and neuronal tissues showing the highest expression levels [15]. RAI1 is thought to function as a transcription factor, based on the presence of a bipartite nuclear localization signal and a zinc finger-like plant homeodomain (PHD) that is conserved in the trithorax group of chromatin-based transcription regulators [12], [17]. It also has homology to the transcription factor TCF20 [16], and contains polyglutamine (polyQ) stretches capable of modulating transcriptional activation [18]. Recently, RAI1 was shown to localize to the nucleus and have transcription factor activity in a neuronal cell line [19]. The *RAI1* promotor region contains several regulatory protein binding sites, including a retinoic acid-responsive element [15]. A variety of mouse studies have identified additional *Rai1* features, including upregulation of *Rai1* in mouse carcinoma cells following retinoic acid treatment [20], localization of the *Rai1* mRNA transcript and protein to neurons suggesting a role in neuronal differentiation [20], and a dosage-dependent role for *Rai1* in the serotonin pathway [21].

To date, only 14 de novo *RAI1* mutations (in 16 patients) have been associated with SMS [9], [10], [11], [12], [13], [14], [22], so more patients need to be evaluated to understand the complete role of *RAI1* in the SMS phenotype. We analyzed 36 patients with SMS features but without a detectable 17p11.2 microdeletion, for variations in *RAI1* and *RAI1* SNP haplotypes. We report 4 *de novo RAI1* mutations, 1 *unclassified* variant, and 5 novel *familial* variants. In addition, we demonstrate for the first time that *RAI1* mRNA expression is decreased in lymphoblastoid cells of SMS patients with the common 17p11.2 deletion, as well as in cells with *RAI1* mutations. We also extensively compare the clinical features of patients bearing the common 17p11.2 deletion with the manifestations of patients having *RAI1* variants, to further delineate which aspects of the SMS phenotype are influenced by *RAI1* expression.

## Results

### Copy Number Analysis

**Figure 1:**
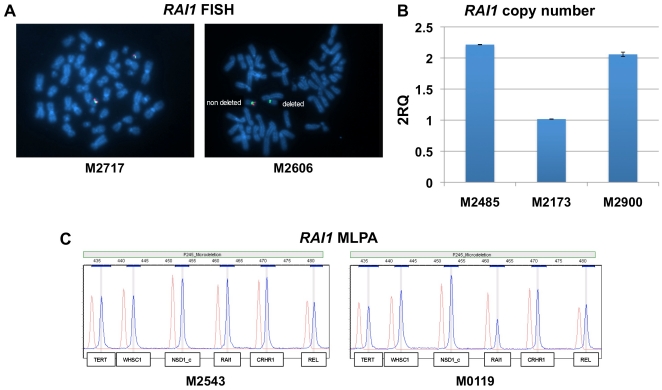
*RAI1* copy number analysis. (A) Representative images of two-color FISH analysis on metaphase chromosomes of lymphoblastoid cells of an SMS patient without (M2717) and with (M2606) the 17p11.2 deletion. The probes were specific for the *RAI1* locus (RP1–253P7; red) and for the chromosome 17 centromere (green). The chromosomes were counterstained with DAPI (blue). (B) Copy number analysis by qPCR using TaqMan primer-probe assays targeting exon 6 of *RAI1* (Hs025670777_s1) and the endogenous control gene *RNaseP*. The comparative Ct method (RQ, relative quantification) was used to determine the *RAI1* gene copy number as shown for a non-deleted patient (M2485), a 17p11.2 deleted patient (M2173) and a non-deleted patient with a *familial RAI1* variant (M2900). (C) Results of MLPA copy number analysis, shown for 6 genes including *RAI1* from the P245-A2 kit. Results are shown for an SMS patient without the 17p11.2 deletion (M2543) and a patient with 17p11.2 deletion (M0119).

Of ∼120 investigated patients with SMS features, 36 were cytogenetically ascertained to have no detectable deletion of 17p11.2. For patients without prior cytogenetic studies, FISH analysis was performed (Figure 1A). Genomic DNA from whole blood was then used to confirm the presence of two *RAI1* alleles in all 36 patients by copy number qPCR (Figure 1B). In selected cases, MLPA analysis confirmed the presence of two *RAI1* alleles (Figure 1C).

### *RAI1* Molecular Analysis

**Figure 2:**
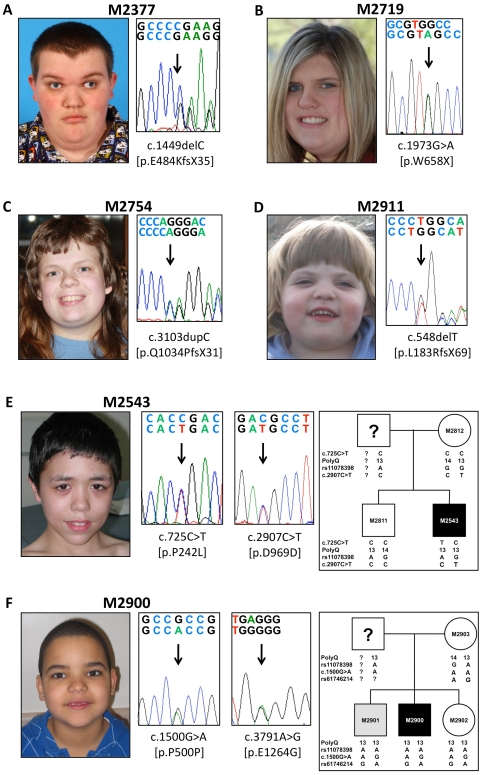
SMS patients and their identified *RAI1* variants. (A) Patient M2377 (pictured at age 20 years) carried the *de novo* frameshift variant c.1449delC. (B) Patient M2719 (pictured at age 17 years) carried the *de novo* nonsense variant c.1973G>A. (C) Patient M2754 (pictured at age 18 years) carried the *de novo* frameshift variant c.3103insC. (D) Patient M2911 (pictured at age 5 years) carried the *de novo* frameshift variant c.548delT. (E) Patient M2543 (pictured at age 14 years) was heterozygous for the c.725C>T and c.2907C>T variants. The pedigree of his family contains the genotypes of his mother (M2812) and his unaffected brother (M2811) for the identified variants as well as the informative SNP rs11078398 and the polyQ repeat sequence. His father's genotype could be partially reconstructed; no paternal DNA was available for sequencing. (F) Patient M2900 (pictured at age 6 years) was heterozygous for the c.1500G>A and c.3791A>G (rs61746214) variants, which were also present in his brother with developmental delay (M2901) and in his unaffected sister (M2902). His family pedigree shows these variants as well as the informative SNP rs11078398 and the polyQ repeat sequence. His father's genotype could be partially reconstructed (no DNA was available).

The *RAI1* coding exons 3, 4, 5 and 6, including their intron-exon boundaries, were sequenced for all 36 undeleted patients and available parents and/or siblings. The identified coding variants (excluding known SNPs) are listed in Table 1. In 4 patients, a severe *RAI1* mutation was identified; we classified these as ‘*de novo*’ variants. Patient M2377 was heterozygous for c.1449delC [p.E484KfsX35], a frameshift mutation leading to a premature stop codon (Figure 2A). This case was previously reported as SMS159 [14]; this variation was absent from parental DNA. Patient M2719 was heterozygous for a novel nonsense mutation, c.1973G>A [p.W658X] (Figure 2B); parental DNA was not available for testing. Patient M2754 was heterozygous for a frameshift mutation, c.3103insC [p.Q1034PfsX31], leading to a premature stop codon (Figure 2C). This case was recently reported as SMS335 [22], and the C-nucleotide at position 3103 was recognized as a frameshift mutation hotspot due to the presence of a heptameric C-tract [22]. This variant was not present in parental DNA. Patient M2911 had an unreported heterozygous frameshift mutation c.548delT [p.L183RfsX69] (Figure 2D). Parental DNA did not contain this variant.

**Table 1:** *RAI1* variants in SMS patients without 17p11.2 deletion identified in the current study.

Patient	Nucleotide change	Protein change	ProbandpolyQ	Father polyQ variants	Mother polyQ variants	Comments
***de novo***						
**M2377**	c.1449delC	p.E484KfsX35	14/14	NA no carrier	NA no carrier	Fig. 2A; SMS159 in [14]
**M2719**	c.1973G>A	p.W658X	13/13	NA^a^	NA^a^	Fig. 2B
**M2754**	c.3103insC	p.Q1034PfsX31	13/14	13/14 no carrier	13/13 no carrier	Fig. 2C; SMS335 in [22]
**M2911**	c.548delT	p.L183RfsX69	14/14	14/14 no carrier	14/14 no carrier	Fig. 2D
***unclassified***						
**M2543**	c.725C>T and c.2907C>T	p.P242L and p.D969D	13/13	NA	13/14 no carrier of c.725C>T carrier of c.2907C>T	Unaffected brother; see Fig. 2E for pedigree
***familial***						
**M2365**	c.3183G>A and c.5653G>A	p.T1061T and p.D1885N	13/13	13/13 carrier of c.3183G>A carrier of c.5653G>A	13/14 no carrier of c.3183G>A no carrier of c.5653G>A	p.D1885N is the first reported variant in exon 4
**M2732**	c.707A>T	p.Y236F	14/14	NA	NA^b^ carrier of c.707A>T	
**M2826**	c.3208G>A and c.4512G>T	p.G1070R and p.L1504L	14/14	NA	13/14 carrier of c.3208G>A carrier of c.4512G>T	Mother is mildly affected
**M2867**	c.3781_3783delGAG	p.del1261E	14/14	14/14 carrier of c.3781_3783delGAG	14/14 no carrier of c.3781_3783delGAG	
**M2900**	c.1500G>A and c.3791A>G^c^	p.P500P and p.E1264G	13/13	NA	13/14 carrier of c.1500G>A carrier of c.3791A>G	Mildly affected brother and unaffected sister; see Fig. 2F for pedigree

NA: DNA was not available.

a This case was classified as ‘*de novo*’ due to pathogenicity of the nonsense mutation, note that parental DNA could not be analyzed.

b Only sequence around c.707A>T available, polyQ was not sequenced.

c Reported rare SNP (rs61746214).

Patient M2543 had a novel heterozygous missense variant, c.725C>T [p.P242L], as well as a novel heterozygous silent variant c.2907C>T [p.D969D] (Figure 2E) and 13 polyQ residues on each allele. The missense variant c.725C>T was not present in his mother (13 and 14 allelic polyQ residues) or brother (13 and 14 allelic polyQ residues). The silent variant c.2907C>T was present in his mother, but not in his brother, indicating that these variants occurred on separate alleles and that the c.2907C>T variant occurred on an allele with 13 polyQ residues that was inherited from his mother. The allele carrying the missense variant c.725C>T was inherited from his father and carried 13 polyQ residues (see pedigree Figure 2E). Since father's DNA was not available, we could not determine whether this variant was *de novo* or paternally inherited, and therefore subgrouped this patient as *unclassified* (Table 1).

In the previously reported patient SMS175 [13], with *RAI1* p.Q1562R, we confirmed absence of the 17p11.2 deletion (M2390, Table S1). However, we did not identify p.Q1562R in whole blood or fibroblast DNA, raising the possibility of mosaicism.

Furthermore, we identified 3 novel heterozygous nonsynonymous (missense) variants, one 3bp deletion and one synonymous (silent) variant (Table 1), all of which were also found in one of the parents. None of these ‘*familial*’ variants were reported SNPs, nor were any identified in our other screened patients or reported in previous *RAI1* sequencing studies [9], [10], [11], [12], [13], [14]. Patient M2365 carried the missense variant c.5653G>A [p.D1885N] as well as the silent variant c.3183G>A [p.T1061T], both of which were identified in his unaffected father but absent from his mother's DNA; they are, therefore, expected to exist on the same allele/in the same haplotype (see also Table S1). Of interest is that p.D1885N is located in *RAI1* exon 4, which is the first reported *RAI1* variant located in this exon.

Patient M2732 and her unaffected mother were heterozygous for the unreported variant c.707A>T [p.Y236F]. Patient M2826 was heterozygous for the novel missense variant c.3208G>A [p.G1070R] as well as a novel silent variant c.4512G>T [p.L1504L], which were both also identified in her mother indicating that they may exist on the same allele/in the same haplotype (see also Table S1). Her mother has a history of learning problems (see Clinical Information S1). Patient M2867 had a novel heterozygous in-frame deletion of 3 bp, c.3781_3783delGAG [p.del1262E] that was also present in her unaffected father and absent in maternal DNA. Patient M2900 carried a heterozygous unreported silent variant c.1500G>A [p.P500P], which was present in the homozygous state in his mildly dysmorphic mother (M2903) and heterozygous in his brother with developmental delay (M2901) and unaffected sister (M2902) (Figure 2F and Clinical Information S1). The paternal DNA was not available for analysis. Further familial molecular studies, including SNP analysis, identified a rare reported SNP, c.3791A>G [p.E1264G] (rs61746214), heterozygous in the proband (M2900), his mother, and his siblings. The more common synonymous SNP c.837G>A [p.Q279Q] (rs11078398) occurred homozygous in the proband and his siblings, and heterozygous in their mother (Figure 2F). These findings indicate that neither the novel silent variant c.1500G>A, nor the identified SNPs are likely to be related to the SMS phenotype in proband M2900.

For the other 26 undeleted SMS patients, no novel *RAI1* variants were detected in the coding region or intron/exon boundaries, other than a variety of reported SNPs (Table S1A).

### Missense Variant Analysis

Table 2 lists all *RAI1* missense variants (detected in this study and those previously reported), as well as nonsynonymous SNPs (indicated with their *rs* identification numbers from dbSNP http://www.ncbi.nlm.nih.gov/snp). Since the pathogenicity of missense mutations is difficult to predict, we analyzed the potential pathogenicity of each variant using different prediction software programs (Polyphen, Panther and PMut). Please note that these are predicted values only, not based on cellular data.

**Table 2:** Severity predictions of missense variants.

Variant type	Nucleotide change	Amino acid change	Polyphen^a^	Panther^a^	Pmut NN output^a^ (Reliability)^b^	Comment
***de novo***						
	c.4685A>G	p.Q1562R	1.639	*−1.77225*	0.6285 (2)	SMS175 in [13]
***unclassified*** c						
	c.725C>T	p.P242L	**2.724**	*−2.22009*	**0.8389** (6)	M2543 our study
	c.5423G>A	p.S1808N	*1.19*	*−2.7097*	*0.1546* (6)	SMS195 in [13]
***familial***						
	c.707A>T	p.Y236F	*0.389*	*−2.55309*	*0.0825* **(8)**	M2732 our study
	c.3634A>G	p.S1212G	*0.297*	*−1.98231*	0.5919 *(1)*	Reported in [11]
	c.5653G>A	p.D1885N	*1.436*	−3.01134	0.3235 (3)	M2365 our study
	c.3208G>A	p.G1070R	1.769	−3.57048	**0.7923** (5)	M2826 our study
	c.3781_3783delGAG	p.del1261E	1.92	-	- (-)	M2867 our study
***SNP***						
rs3803763	c.269C>G	p.G90A	*0.124*	*−1.00625*	*0.2039* (5)	
rs11649804	c.494C>A	p.P165T	**2.274**	*−2.6508*	0.407 *(1)*	
rs61746214	c.3791A>G	p.E1264G	**2.145**	*−2.8526*	**0.7363** (4)	M2900 our study

a Effect on the protein: Benign, *italic print*; Ambiguous, underlined; Deleterious, **bold print**.

b Reliability score: Poor, *italic print*; Medium, underlined; Good, **bold print**.

c Parental DNA was not available for testing.

The identified p.P242L missense variant (patient M2543) has a high probability to be deleterious predicted by at least 2 programs. The previously published *RAI1* missense mutations p.Q1562R (SMS175) [13] and p.S1808N (SMS195) [13] were predicted to be benign or ambiguous deleterious by all 3 prediction programs. Interestingly, a recent report demonstrated that neither of these two variants impair RAI1 nuclear localization or transcription factor activity [19], suggesting that these variants may not cause the SMS phenotype, or that other factors (post-translational modifications, interactions) related to these mutations may induce their SMS phenotype.

The familial missense variants p.Y236F, p.S1212G, p.D1885N, and p.del1261E were predicted to be benign overall, based on at least 2 prediction programs (except for p.del1261E, which could only be analyzed by the Polyphen program, Table 2).

Of the 3 nonsynonymous SNPs, p.G90A (rs3803763) was predicted to be benign, p.P165T (rs11649804) has variable predictions, but p.E1264G (rs61746214) was predicted by Pmut and Polyphen to be deleterious and warrants further research.

*RAI1* is highly polymorphic; more than 30 SNPs are reported in the coding region in dbSNP. All identified variants of our molecular analyses are listed in Table 3. For each variant, the minor allele frequency (MAF; the frequency of the SNP's less frequent allele in a given population) reported in dbSNP, as well as the MAF calculated from our study are indicated in Table 3 (see also Table S1 for allele distributions). Our SMS patient contingent was of Caucasian origin (except patient M2900 who was Hispanic, and M2543 who had a mother of Indian descent). For most variants, the MAF identified in our study is similar to that reported in dbSNP, except for three variants, rs8067439 and rs3803763, which occurred more frequently in our SMS cohort and rs35686634, which occurred less frequently in our SMS cohort (gray highlighted in Table 3).

**Table 3:** Minor allele frequencies (MAF) of *RAI1* variants.

dbSNP ID^a^	Nucleotide position^b^	Nucleotide change^c^	Protein change	MAF dbSNP	MAF Current study^d^
rs3803763	c.269	C>G	p.G90A	0.357^e^	**0.71**
rs11649804	c.493	C>A	p.P165T	0.242^f^	0.29
rs11078398	c.837	A>G	p.Q279Q	0.417^e^	0.64
polyQ	c.832–873		9–15 Q		
rs8067439	c.1992	G>A	p.P664P	0.017^f^	**0.79**
rs61746214	c.3791	A>G	p.E1264G	NR^g^	0.01
rs4925112	c.4311	T>C	p.P1437P	0.034^f^	0.03
rs35686634	c.4530	C>T	p.P1510P	0.103^e^	**0.03**
rs3818717	c.5601	C>T	p.I1867I	0.298^f^	0.36
-	c.707	A>T	p.Y236F	-	0.01
-	c.725	C>T	p.P242L	-	0.01
-	c.1500	G>A	p.P500P	-	0.01
-	c.2907	C>T	p.D969D	-	0.01
-	c.3183	G>A	p.T1061T	-	0.01
-	c.3208	G>A	p.G1070R	-	0.01
-	c.3815	GGA>_	p.del1261E	-	0.01
-	c.4512	G>T	p.L1504L	-	0.01
-	c.5653	G>A	p.D1885N	-	0.01

a dbSNP: http://www.ncbi.nlm.nih.gov/snp.

b Numbering is based on cDNA (NM_030665), with +1 corresponding to the A of the ATG initiation codon.

c Major allele>Minor allele.

d **Bold print**: significant differences from dbSNP.

e Determined on AGI_ASP normal panel (Coriell Repositories, Camden, NJ).

f Determined on HAPMAP CEU population.

g NR, not reported.

### SNP Haplotype Analysis

We attempted to reconstruct the haplotype for each patient by assigning the variant nucleotides to each allele, using all sequencing data including sequences from available family members. For most patients, the listed haplotypes are the only possible combination of variants; for other patients the haplotype is the most likely prediction (Table S1). We prioritized the presence of a ‘common haplotype’ allele (Haplotype H1 in Table S1), and then assigned the nucleotides of the second allele. These analyses revealed various allelic haplotypes among 72 studied alleles, with one predominant haplotype existing on 44% of the alleles (H1: 32 of 72 alleles, yellow highlighted in Table S1), one moderately common haplotype existing on 15% of alleles (H2, green highlighted) and several rare haplotypes, with existence ranging from 3%–7% of alleles, and 11 unique haplotypes (u, white background, 17%) (Table S1).

### *RAI1* mRNA Expression

**Figure 3:**
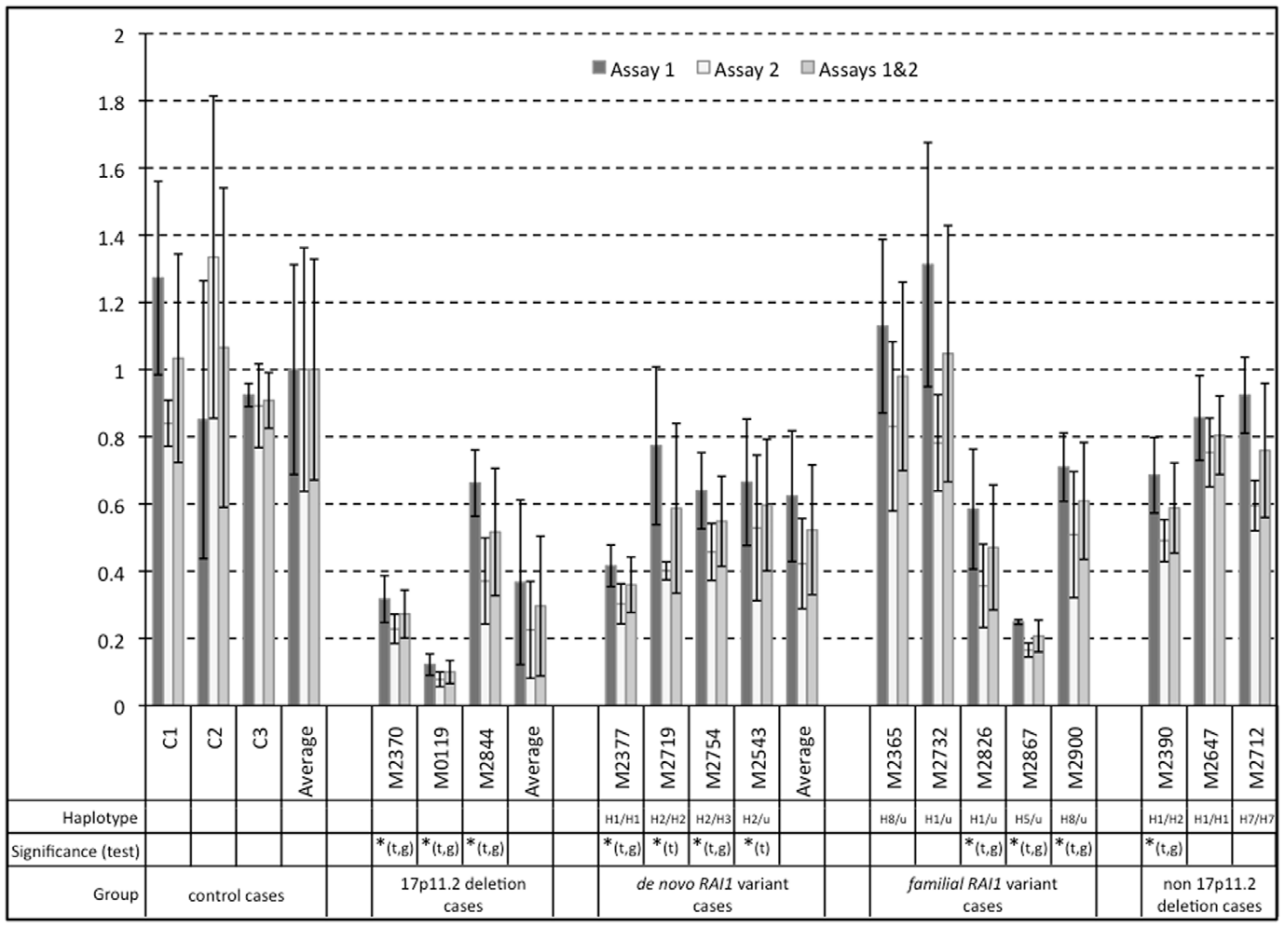
*RAI1* mRNA expression in lymphoblastoid cells. RNA extracted from lymphoblastoid cells from SMS patients in 4 subgroups: cases with common 17p11.2 deletion, *de novo RAI1* variants (including the ‘unclassified’ variant M2543), *familial RAI1* variants, and non-17p11.2 deleted without identified *RAI1* variants, as well as from 3 control cell lines were used for *RAI1* mRNA expression analysis by qPCR. Two Taqman primer-probe assays were used per sample (assay 1 and assay 2). Displayed values represent the relative quantification (RQ) compared to the average of all control assays (set to 1). *: Average RQ of the sample is statistically different (p<0.05) from the average of all control cases (t: using the ANOVA post hoc Tukey-Kramer test; g: using the ANOVA post hoc Games-Howell test).

*RAI1* mRNA expression levels were determined by qPCR on RNA isolated from lymphoblastoid cells (Figure 3). SMS patients with the common 17p11.2 deletion (M2370, M0119, M2844; haploinsufficient for *RAI1*) had significantly (p<0.05) lower expression of *RAI1* mRNA, with an average of ∼30% of control. In addition, all patients with *de novo RAI1* variants displayed significantly decreased *RAI1* expression (p<0.05 by at least one statistical test) to about 52% of normal; cells from patient M2911 were not available. Decreased *RAI1* expression was not only determined in cells with *RAI1* frame-shift and nonsense mutations (36% in M2377, 59% in M2719, and 55% in M2754), but also in the patient with a missense mutation (60% in M2543).

Expression levels varied among the *familial RAI1* variants (M2365, M2732, M2826, M2867, M2900) and three selected non-deleted cases without novel *RAI1* variants (M2390, M2647, M2712). In this group, *RAI1* expression varied from normal and non-significant (98% in M2365, 104% in M2732, 80% M2647, 76% in M2712), to moderately but significantly (p<0.05) decreased (61% in M2900 and 59% in M2390), to significantly severely decreased (47% in M2826 and 21% in M2867). An alternative normalizing gene (instead of β-actin), *G6PC3* was used for qPCR on selected mRNA samples from each group, demonstrating that normalizing to a control assay with a similar threshold cycle (Ct) as the *RAI1* assays provided comparable results to using β-actin as normalizing gene (Figure S1).

Since genomic copy number variations are a concern when using EBV transformed cells [23], [24], we also performed MLPA analysis on genomic DNA from all lymphoblastoid cell lines (Figure S2). We verified that all cell lines had two alleles for *RAI1*, except for the 17p11.2 deleted cases (M2370, M0119, M2844), who were confirmed to have one copy of the 17p11.2 genes *RAI1*, *LRRC48*, and *LLGL1*. Cell lines M2365, M2370 and M2867 showed a variety of abnormal copy number variations outside the 17p11.2 region (Figure S2).

We were unable to analyze the translated amounts of RAI1 protein, since the commercially available RAI1 antibodies that we tested (RAI-1 C-14 from Santa Cruz Biotechnology and LS-C46854 from LifeSpan) did not yield a RAI1 signal by western blotting of lymphoblastoid cell extracts.

### Clinical Analysis

Detailed clinical descriptions of the cases with *de novo* and *familial RAI1* variants are provided in the Clinical Information S1. Comparison of clinical features of our *de novo* subgroup with previously reported *RAI1* mutation and 17p11.2 deletion cases is summarized in Table S2, and evaluated below. We provide clinical comparison data with and without the ‘*unclassified*’ variant M2543 included in the ‘*de novo*’ cohort, and mention where he is an outlier. We did not analyze the *RAI1 familial* variants as a discrete phenotypic group, partly due to the heterogeneity of their *RAI1* levels (Figure 3).

#### Growth parameters

Birth parameters for *de novo RAI1* variant cases included term (mean 39.6±2.2 weeks) delivery and appropriate-for-gestational age (AGA) birth weights and lengths, consistent with published data for both 17p11.2 deletion (∼80% term) [25] and *RAI1* mutation cases [11], [12], [13], [14]. Among the *de novo RAI1* subgroup, four patients had current weights >98^th^ centile (obese) and lacked short stature (<5^th^ centile), including the youngest (M2911, 5y). Only M2543 appeared to be an outlier in this group with weight in the normal range and short stature (height <2^nd^ centile). Head circumferences were normal for three (M2719, M2754, M2911) and >95^th^ centile for one (M2377); microcephaly (OFC<2^nd^ centile) was observed only in M2543, the potential outlier.

**Figure 4:**
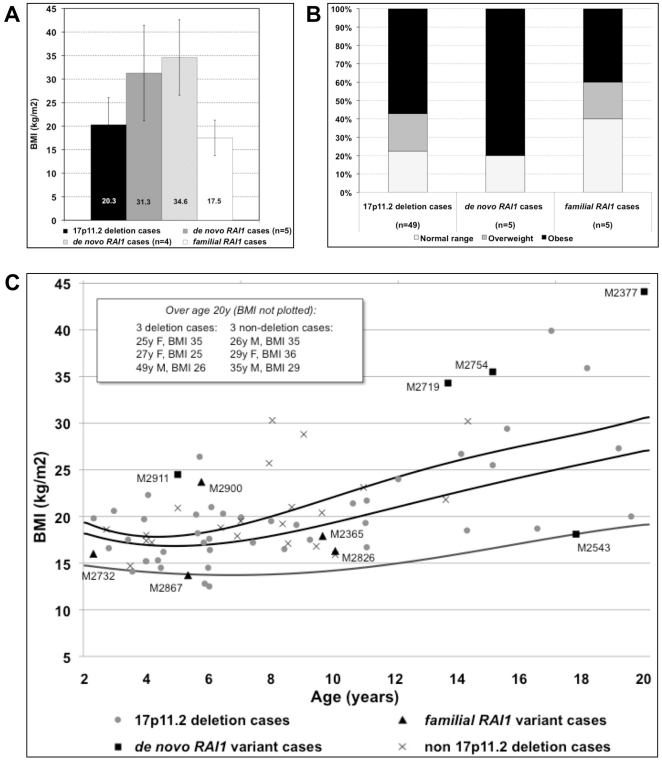
Body mass index (BMI) analysis of SMS patients. Comparison of BMI (kg/m2) for common 17p11.2 deletion cases (n = 49), and cases with *de novo* (n = 5, including unclassified variant M2543) or *familial* (n = 5) *RAI1* variants. (A) The mean BMI for each subgroup. The value for the *de novo RAI1* group was calculated with and without the outlier *unclassified* case (M2543) who carried an *RAI1* missense variant. (B) Frequency of body description type (*normal*, *overweight*, or *obese*) based on BMI values considering age (as plotted in (C)) and gender. Interpretation of BMI levels for age 2–20 years: underweight, <5^th^ percentile; normal range, 5^th^–85^th^ percentile; overweight, 85^th^–95^th^ percentile; and obese, >95^th^ percentile. For adults: underweight, BMI below 18.5; normal range, BMI 18.5–24.9; overweight, BMI 25–29.9; and obese, BMI 30 and over. (C) Comparison of BMI by age for subjects 2–20 years of age. BMI percentile curves (5^th^, 85^th^ and 95^th^) for ages 2–20 years were extracted from growth data from the Centers for Disease Control and Prevention. BMI values are not plotted for 6 subjects over age 20 years; 3 with common deletions and 3 without deletions or *RAI1* mutations (their values are displayed in upper left of the figure).

The mean BMI for the *de novo* group (n = 5; 31.3±10.1 kg/m2) was significantly higher than for the SMS 17p11.2 common deletion group (n = 49; 20.3±5.8 kg/m2) by the two-tailed unpaired t-test (t = 3.7, df = 51; p<0.0005 (Figure 4A and 4B). BMI values above 25.0–29.9 kg/m2 are considered overweight and ≥30 kg/m2 are consistent with obesity as defined by the Centers for Disease Control and Prevention (http://www.cdc.gov/growthcharts/). Based on this classification, except for patient M2543 (the missense variant outlier), all *de novo RAI1* cases are obese, including the youngest (M2911, 5y) in contrast to 57% (28 out of 49) of common 17p11.2 deletion cases (Figure 4B and 4C). The observed frequency distribution of body types (Figure 4B) by subgroup was not statistically significant (Chi square 6.0; p = 0.42). Age was significantly correlated to BMI for the entire study group (Spearman's rho 0.60; p<0.0001) (Figure 4C). However, analysis by subgroup showed a significant correlation between BMI and age for only the two largest subgroups: the common deletion cases (n = 49; Spearman's rho 0.576; p<0.0001) and the non 17p11.2 deletion cases without *RAI1* variants (n = 24; Spearman's rho 0.585; p = 0.005). Both the *de novo* (n = 5) and *familial* (n = 5) *RAI1* variant subgroups were non-significant (Figure 4C).

#### Neurobehavioral features

cases included: problems with food intake and/or food foraging (5/5 *de novo* cases); nail yanking (4/5 *de novo*; not M2543 outlier); and to a lesser extent anxiety/mood shifts (5/5 *de novo*; including M2543 outlier). Speech delay was seen less frequently in the *de novo* group (3/5) compared to published deletion cases (>90%) and remains close to prior studies (70%) [9], [10]. All subjects without the 17p11.2 deletion and SMS diagnosis in our study cohort had neurobehavioral features that overlap with deletion cases (Table S2), likely reflective of referrals for study by experienced clinicians. Behavioral features that might distinguish the *de novo* subgroup from common deletion.

#### Concordant features with 17p11.2 deletion cases

Hypotonia, frequent otitis media, ocular anomalies, dental anomalies, hoarse voice, and brachydactyly occurred in our *de novo RAI1* cases with frequencies consistent with published 17p11.2 deletion cases (Table S2). Scoliosis was seen in 3/5 *de novo* (M2377, M2719, M2754) cases, consistent with published frequencies for common deletion (40–70%) and *RAI1* mutation (36%) cases [9], [10]. Psychomotor delay, sleep disturbance and typical behavioral features occurred in over 80% of the *RAI1* cases (Table S2). Hearing loss occurred in 4/4 *de novo* cases tested compared with 60–79% for 17p11.2 deletions and 10–25% for published *RAI1* mutation cases [3], [9], [10]. Our two oldest cases (M2377 and M2754) had sensorineural hearing loss (SNHL).

#### Discordant features with 17p11.2 deletion cases

Seizures occurred in all but one of the *de novo* cases, compared with only 11–30% for deletion cases and 17% for published *RAI1* mutation cases [4], [9], [10]. Obstructive sleep apnea (OSA)/tonsillectomy/adenoidectomy were more prevalent in the *de novo* cases (5/5) compared to our deletion cases (50%). Cardiovascular and renal abnormalities were not documented in any *de novo* cases, consistent with prior reports [3], [9], [10]. While structural genitourinary anomalies were absent, issues of incontinence and/or nighttime enuresis were common, and frequent urinary tract infections occurred in all three females in the *de novo* subgroup. Other genital findings included hypogonadism (M2377) and labial adhesions (M2911). With the exception of a bifid uvula documented in M2719, facial clefts were absent. Immunological abnormalities were not identified. In addition, failure to thrive (FTT)/feeding issues were less frequent (3/5) in *de novo RAI1* mutation cases compared to deletion cases (19/19) [26]. Both gastroesophageal reflux disease (GERD) and constipation issues occurred in *de novo* cases (2/5), but less frequently than reported for deletion cases [27].

## Discussion

In most microdeletion syndromes, haploinsufficiency of more than one gene underlies the phenotype [28], [29]. In others, such as Alagille syndrome (deletion of 20p12; OMIM 118450) or Rubinstein-Taybi syndrome (deletion of 16p13.3; OMIM 180849), haploinsufficiencies of a single gene (*Jagged1* (*JAG1*) or *CREBBP*, respectively) accounts for all the characteristic features [30], [31], [32]. In still other syndromes, haploinsufficiency of one gene in the deleted region explains only some specific feature(s); haploinsufficiency of the elastin gene accounts for the cardiac defects in Williams-Beuren syndrome (deletion of 7q11.23; OMIM 194050) [33] and haploinsufficiency of the *LIS1* gene explains the lissencephaly of Miller-Dieker syndrome (deletion of 17p13.3; OMIM 247200) [34], [35].

SMS is considered a microdeletion syndrome in which haploinsufficiency of multiple genes underlies the phenotypic features [3], [5], [9]. However, heterozygous mutations in *RAI1* have been identified in clinically typical SMS patients without detectable 17p11.2 deletions. This raises the issue of how *RAI1* haploinsufficiency influences *RAI1* RNA transcription, and which clinical features of SMS result from *RAI1* haploinsufficiency.

According to BioGPS (Human Gene Atlas U133A; http://biogps.gnf.org) [36], [37], *RAI1* is expressed in 84 different human tissues, including B-lymphoblasts. We employed lymphoblastoid lines to assess *RAI1* expression in our patients, after ruling out copy number variations due to the immortalization process by MLPA (Figure S2).

Our results indicated that haploinsufficiency of *RAI1* (through deletion of 17p11.2) results in a greater than 50% decrease in *RAI1* expression (Figure 3). Other factors, likely deleted ancillary genes in 17p11.2, may influence *RAI1* expression to decrease below the expected 50% level. For example, it was recently demonstrated that *HDAC4* haploinsufficiency (on chromosome 2q37) decreased *RAI1* mRNA expression to lower than 50% levels [38]. All our 4 patients with *de novo RAI1* variants had approximately 50% decreased *RAI1* levels (Figure 3), likely due to RNA decay of the nonsense (M2719) and frame-shift mutated (M2377, M2754, M2911) alleles. These findings are consistent with *RAI1* expression levels reported for a haploinsufficient *RAI1* mouse model [39]. Our ‘*unclassified*’ patient M2543 carried a missense (and a silent) *RAI1* variant and displayed decreased *RAI1* expression; whether his RNA expression level is directly related to these variants is unknown. We found no obvious correlation between *RAI1* haplotype (Table S1) and RNA expression (Figure 3).

Surprisingly, selected SMS patients without truncating *RAI1* mutations displayed significantly decreased *RAI1* expression in both the *familial* variant group (47% in M2826; 21% in M2867, and 61% in M2900) and in a non-deleted case (59% in M2390; SMS175 in ref. [13]) (Figure 3). These reduced levels may help explain their clinical SMS-like phenotype, supported by recent data of patients mutated in *HDAC4*, showing impaired *RAI1* mRNA expression (without *RAI1* mutations) and exhibiting a SMS-like phenotype [38]. In addition, sequence variations in non-coding *RAI1* exons 1 and 2, the 3′untranslated region (UTR), or in (conserved) intronic regions may underlie the decreased *RAI1* levels. In addition, *RAI1* expression may be affected by (epigenetic) modifiers within or outside the common 17p11.2 deletion region; environmental or physiological factors may also play a role [40]. These findings emphasize that RAI1 expression is a promising genetic target for development of therapeutic interventions for SMS.

In evaluating the clinical features of SMS in relation to molecular results, we found that a high BMI and obesity are characteristic of the *de novo RAI1* variant cases (4/5), as previously reported (6/9 or 67%) [3]. In our common deletion cases, the frequency of obesity (28/49 or 57%; Figure 4B) was higher than previously reported (4/31 or 13%) [3], perhaps reflecting age at assessment and pubertal status. In the study by Edelman et *al.* [3], median assessment ages were 15 years (*de novo RAI1* mutation cases) and 8 years (17p11.2 deletion cases), compared to 15 years (*de novo RAI1* cases) and 14 years (17p11.2 deletion cases) in our analysis. A trend toward obesity in common deletion cases was reported [25], beginning around age 9 years, coinciding with pubertal onset, and reaching >95^th^ centile for weight in teenage years to adulthood.

Past reports suggest that several features occur less often or are less severe among *RAI1* mutation cases compared to common 17p11.2 deletion cases. These include infantile hypotonia, short stature, speech and motor delay, hearing loss, frequent otitis media, and structural cardiac and renal defects [3], [9], [10]. Consistent with previously published reports, our *de novo RAI1* variant cases (Table S2) were less cognitively impaired (mild intellectual disability), lacked short stature (except for outlier M2543), and had normal cardiac and renal structure. While delays in growth (height/weight) in early childhood were previously recognized for *de novo RAI1* mutation cases [13], the frequency of failure to thrive (FTT) and feeding issues in infancy has not been documented. In our study group, FTT and early feeding issues occurred less frequently among *de novo RAI1* variant cases (3/5) compared to reported for SMS deletion cases (19/19; 100%) [26].

We identified several features that occurred more frequently in our *de novo RAI1* variant cases than in previously reported cases. Infantile hypotonia was documented more often in our *de novo* subgroup (5/5) than previously reported (44%–61%) [3], [9], [10]. Seizures (with/without EEG abnormalities) also occurred more frequently in our *de novo* (4/5) group than previously reported (17%) [9], [10]. As expected, behavioral features occurred across all subgroups, reflecting syndrome-specific features that include sleep disturbance and various maladaptive and self-injurious behaviors. Interestingly, only 3/5 of our *de novo RAI1* variant cases demonstrated the characteristic “self-hug”, which is more consistent with the reported rate for deletion cases (50–80%) compared to the 100% (9/9) previously reported for *RAI1* mutation cases [3], [9], [10]. As expected, sleep disturbance was universal, but we also documented increased symptoms of OSA and/or T&A for our *de novo* (5/5) group. In addition, anxiety issues, rapid mood shifts and emotional lability were present in 5/5 of our *de novo RAI1* variant group, raising future research questions concerning the role of *RAI1* in neurodevelopment.

Only two clinical features (Table 4) demonstrated a significant relationship to *RAI1* mRNA levels, i.e., ocular abnormalities (Mann-Whitney Z = −2.35; p = 0.0188) and object insertion (Mann-Whitney Z = −2.21; p = 0.03). Some ocular abnormalities, either strabismus (2/4), esotropia (3/4), or hyperopia (1/4), were present in all our *de novo RAI1* cases; this frequency is higher than previously appreciated [9], [10]; and more consistent with common 17p11.2 deletion cases (Table 4 and Table S2). Although object insertion was significantly associated with lower *RAI1* expression levels (Table 4), this behavioral feature may reflect a bias of ascertainment since it would lead to referral for *RAI1* mutation analysis of suspected SMS non-deleted cases.

**Table 4:** Comparison of mean *RAI1* levels based on presence/absence of phenotypic features.

Group (patient)	Age (years)	mRNA level^a^	BMI (kg/m2)^b^	Body description^b^	FTT/feeding issue (infancy)	Ocular	Dental	Hearing loss	Hoarse voice	Scoliosis/vertebral anomalies	Brachydactyly	Immune abnormalities	Other infections	Seizures +/− abnormal EEG	Genital anomalies/pubertal delay	Speech delay	OSA and/or T&A	Food intake	Self-injurious behavior^c^	Onycholtillomania (nail yanking)	Polyembolokoiloma-nia (object insertion)
*de novo RAI1 cases*																					
M2377	20	36%	44	obese	n	**y**	y	**y**	**y**	y	y	**n**	y	y	y	n	y	**y**	y	**y**	y
M2719	13	59%	34	obese	n	**y**	n	**y**	**y**	y	y	**n**	n	y	n	n	y	**y**	y	**y**	y
M2754	15	55%	36	obese	y	**y**	y	**y**	**y**	y	n	**n**	y	y	n	y	y	**y**	y	**y**	n
M2911	5	ND	25	obese	y	**y**	y	**y**	**y**	n	y	**n**	y	n	y	y	y	**y**	y	**y**	y
M2543^d^	17	60%	18	normal	y	**y**	y		**y**	n	y	**n**	n	y	n	y	y	**y**	y	**n**	n
*familial RAI1 cases*																					
M2365	9	98%	18	overwt	y	n	n	y	y	n	y	y	y	y	y	y	n	n	y	y	n
M2732	2	105%	16	obese	y	y	y	n	NV	n	n	y	n	n	n	y	y	n	y	n	n
M2826	10	47%	16	normal	y	y	y	y	n	y	y	n	y	y	y	y	y	y	y	n	y
M2867	5	21%	14	normal	y	y	n	n	n	n	n	y	y	y	n	y		n	n	n	y
M2900	6	61%	24	obese	n	n	y	n	y	n	y	y	y	y	n	y	y	y	y	y	y
*non-deleted cases*																					
M2390	11	59%	23	overwt	n	y	y	n	y	n	n	y	y	y	n	n	n	n	y	y	y
M2647	4	80%	17	obese	y	n	n	n	n	n	n	n	y	n	n	y	y		y	n	y
M2712	7	76%	20	obese	y	n	y	n		n	n	y	y	y	y	y	y	n	y	y	n
*common del 17p11.2 cases*																					
M0119	16	10%	19	normal	y	y	y	y	y	n	y	y	y	n	n	y	n	y	y	y	y
M2844	4	52%	15	obese	n	y	y	n	n	n	y	n	n	y	n	y	n	n	y	n	y
M2370	14	27%	27	obese	n	y		n	y	n	y	y	n	y	n	y	y	n	y	y	y
*Mann-Whitney* Z-value^e^					−0.82	−2.35	−0.57	−0.90	−0.46	−1.04	−1.18	−0.35	−0.24	−0.72	−0.39	−0.68	−0.42	−0.83	−1.39	−0.35	−2.21
*Sign. of RAI1 level vs. feature* f					ns	0.02	ns	ns	ns	ns	ns	ns	ns	ns	ns	ns	ns	ns	ns	ns	0.03

Abbreviations: n = not present; y = present; blank = insufficient data for determination; BMI, Body Mass Index; FTT, failure to thrive; NV, non-verbal; OSA, obstructive sleep apnea; overwt, overweight; T&A, tonsillectomy and/or adenoidectomy. **Bold underlined**: specific for *de novo RAI1* cases.

a Mean mRNA levels as calculated in Figure 3.

b BMI and body description based on BMI for age determined using http://www.halls.md/body-surface-area/bsa.htm.

c Self-injurious behavior is present in all but 1 subject (M2867); hypotonia and sleep disturbance are present in all cases, excluding these features from statistical analysis.

d Patient M2543 is an ‘*unclassified*’ case, analyzed here with the ‘*de novo*’ group as described in the text.

e Significance of *RAI1* mRNA level for presence (y) or absence (n) of each clinical feature determined by nonparametric Mann-Whitney Z-value.

f p values as calculated by nonparametric Mann-Whitney test; significant p<0.05; ns, non-significant p>0.05).

While not significantly associated with *RAI1* level, several clinical features (Table 4) may differentiate cases with *de novo RAI1* variants from the other sub-groups. All four *de novo* cases tested demonstrated hearing loss in contrast to 25% (2/8) previously reported, the role of *RAI1* in hearing abnormalities is unknown [3]. Since the *Myosin 15A* (*MYO15A*) gene, located in the 17p11.2 SMS critical region, was implicated as a candidate gene for the hearing abnormalities of SMS [41], it is of interest to explore *MYO15A* expression in SMS patients as well as the role of *RAI1* in *MYO15A* expression. The absence of immunologic abnormalities (Table 4) in our *de novo* cases, versus the increased frequency reported for deletion cases (23–50%) [42], [43], suggests that a gene other than *RAI1* may regulate immune involvement in SMS. The *TNFRSF13B* gene, located in 17p11.2, encoding the transmembrane activator and CAML interactor (TACI) protein, was proposed as a candidate for the immune abnormalities, including reduced IgA levels in SMS patients [43], [44]. The presence of a hoarse voice occurred in all our *de novo* cases, but was not significantly related to *RAI1* expression levels. Furthermore, no apparent correlation between specific clinical features and *RAI1* haplotype or polyQ repeat length (Table S1) could be identified. It is of interest to note that a spina bifida occulta (SBO) variant occurred in one *de novo* (M2377) and one *familial* (M2826) case, both with *RAI1* levels <50%.

Failing to document a direct correlation between *RAI1* level and most features may reflect the small sample size and/or bias introduced by features leading to referral for suspected SMS in non 17p11.2 deletion cases. It is also possible that our group categorization of subjects reflects an arbitrary designation. The *familial* variants were not analyzed as a discrete clinical subgroup due to the heterogeneity of their *RAI1* levels. No feature(s) emerged to distinguish the two females with low *mRAI1* levels (M2826, 47%; M2867, 20.7%) from others in the *familial* subgroup. *Familial* cases may be similar to non-deletion cases without *RAI1* variants or, in cases where family members present with subtle overlapping symptoms, further familial analysis of *RAI1* expression could shed more light on the role of the *RAI1* variants. For example, our case M2900, the mother and developmentally delayed brother both showed features not observed in his cognitively normal sister (see Clinical Information S1), yet all have the same *familial RAI* variant. Such cases reiterate the importance of family studies to verify the inheritance of the variant. We classified M2543, who has a severe missense *RAI1* variant, as ‘*unclassified*’ since his father was unavailable for genetic testing. Reasons to analyze the clinical and molecular findings of M2543 with the ‘*de novo*’ subgroup were the severity of his missense variant (Table 2) and his decreased *RAI1* expression level of 60% (Figure 3, Table 4), although this level was the highest in the *de novo* group. On the other hand, M2543 appears to be an outlier from the *de novo* group for several clinical features, including short stature (<5^th^ centile), normal BMI (non-obese), less characteristic facial appearance (See Figure 2E) with OFC at 2%, and increased level of cognitive impairment with significant speech delay.

Our clinical analysis as well as our large group of undeleted patients without detected *RAI1* variants (26 patients, Table S1) indicates that other genes may be involved in the complex SMS phenotype. A future approach would be to determine *RAI1* expression levels in this group of non-deleted cases as well as expression levels for other genes in the 17p11.2 critical region that have been implicated to play a role in some SMS features, including *MYO15A* (hearing) [41], *TNFRSF13B* (immune) [43], *PEMT* (fatty liver) [45], and *ALDH3A2* (dry skin) [46]. We realize that defects in other chromosomal regions could be present in these patients, which will be pursued by whole genome array studies, as recently described for other SMS patients [47].

An ancillary dividend of this study is our analysis of the pathogenicity of *RAI1* variants. It is reasonable to assume that the nonsense and frame-shift *RAI1* variants would lead to nonsense-mediated decay [48]; the resulting haploinsufficiency of *RAI1* could lead to the SMS phenotype, as suggested for patients with the common 17p11.2 deletion [7], [49]. However, it remains unknown how *RAI1* missense mutations can underlie the SMS phenotype. Our haplotype analysis showed that *de novo* and *familial RAI1* variants did not appear to occur on a preferred haplotype (Table S1). Our pathogenicity assessments of *RAI1* missense variants (Table 2) showed that p.P242L (M2543) was predicted to be deleterious by at least 2 programs. However, before calling this variant a mutation, paternal DNA (not available to us) should be analyzed, and we therefore sub-grouped this patient as ‘*unclassified*’. Two previously published missense variants, p.Q156R and p.S1808N (SMS175 and SMS195 respectively [13]), were predicted to be benign or ambiguously deleterious by all 3 prediction programs (Table 2), and did not influence RAI1 nuclear localization or transcription activity [19]. This warrants further research regarding the pathogenicity of these two variants.

Most *familial* missense variants were predicted to be benign by at least 2 prediction programs. These predictions, in cases where the carrier parents are apparently unaffected, render these variants unlikely to be disease causing. The *familial* variant p.G1070R (patient M2826) was predicted to be ambiguous and deleterious. This variant was also present in the patient's mother, who had learning problems (see Clinical Information S1), and may play a role in the severe clinical phenotype of the patient and mild symptoms in her mother.

One of the three nonsynonymous *RAI1* SNPs, p.E1264G (rs61746214), was predicted to be deleterious, but familial analysis showed that this variant may not be disease causing in patient M2900 (Figure 2F). The allele frequency of rs61746214 is not reported in dbSNP; we only identified this allele (of 72 analyzed) in patient M2900. Since this individual was the only Hispanic in our study, the frequency of rs61746214 should be determined in the Hispanic population.

In sum, identification of additional *de novo RAI1* cases is required to further delineate phenotypic heterogeneity in this SMS subgroup. Our study adds two newly ascertained *de novo RAI1* mutation cases, one unclassified case, and provides further assessment of two previously reported cases (M2377/SMS159 [14] and M2754/SMS335 [22]). As noted, early published *RAI1* mutation cases may reflect a bias of ascertainment due to the striking phenotypic similarity to deletion cases, especially with respect to the physical and neurobehavioral features of the syndrome that become more evident with age. Cases suspected to have SMS, but without a 17p11.2 deletion, should prompt consideration of *RAI1* mutation analysis, if their features include AGA term birth, childhood onset obesity (increased BMI for age), ocular abnormalities, hoarse voice, middle ear dysfunction and hearing loss, and behavioral aspects, especially self-injurious behavior, nail damage, and problems regulating food intake (i.e., insatiable appetite), in the absence of immunological abnormalities and cardiovascular or renal structural anomalies.

## Materials and Methods

### Ethics Statement

All patients were enrolled in NIH clinical protocol 01-HG-0109 approved by the National Human Genome Research Institute (NHGRI) institutional review board to evaluate the clinical and molecular manifestations of Smith-Magenis syndrome (www.clinicaltrials.gov, NCT00013559). Written informed consent was obtained from each patient or their parents. All clinical investigations were conducted according to the principles expressed in the Declaration of Helsinki.

### Study Group

Since universally agreed minimum diagnostic criteria for SMS are lacking, patients were included based on the clinical impression of experienced clinicians of clustering of features (i.e., facial appearance, unusual sleep pattern, behavioral and developmental concerns) suggestive of SMS.

Clinical data for participating subjects were derived from chart review of medical records and genetic evaluations at the NIH or offsite. Descriptive statistics including weight and height percentiles and body mass index (BMI) were calculated using an on-line body surface area calculator for medication doses (http://www.halls.md/body-surface-area/bsa.htm). For statistical analysis, growth parameters of ‘*de novo*’ and ‘*familial*’ *RAI1* variants were compared to a common 17p11.2 SMS deletion group of 49 patients (30 female/19 male; mean age 9.6±8.4 years; range 1.4 to 49 years), also evaluated under our NIH clinical protocol.

Peripheral blood was collected from the patients and employed for extraction of genomic DNA and for Epstein Barr Virus (EBV) immortalization of B-lymphocytes, using standard protocols. Primary cultures of epidermal fibroblasts were obtained from selected patients from a forearm skin biopsy or from tissue procured from a surgical sample and cultured as previously described [50].

### Cytogenetic Analysis

A subset of patients enrolled in our protocol had prior fluorescent *in situ* hybridization (FISH) results from studies performed by outside laboratories. For most patients without prior cytogenetic studies, we performed FISH with DNA probes specific for the *RAI1* locus (RP1–253P7), as well as a distal SMS-REP (RP11–416I2) and a proximal SMS-REP (RP5–836L9) 17p11.2 probe, as described [51].

### Copy Number Analysis

Genomic DNA (gDNA) of all enrolled patients was subjected to *RAI1* copy number analysis by quantitative PCR (qPCR). For qPCR, TaqMan primer-probe assays targeting exon 6 of *RAI1* (Hs025670777_s1) and the endogenous control gene *RNaseP* were purchased from Applied Biosystems (Foster City, CA). gDNA samples of SMS patients, along with control samples, were PCR-amplified in triplicate as described [52] for both assays on an ABI PRISM 7900 HT Sequence Detection System (Applied Biosystems). The comparative Ct method was used to determine the *RAI1* gene copy number [52], [53], [54]. For copy number analysis by multiplex ligation-dependent probe amplification (MLPA), the P245-A2 Microdeletion Syndromes-1 kit was employed, which includes a probe for *RAI1*, following the manufacturer's recommendations (MLPA® MRC-Holland, Amsterdam, The Netherlands). Genescan-ROX 500 (Applied Biosystems) was added to the reaction mixtures to facilitate estimation of fragment sizes. MLPA fluorescent PCR products were separated on an ABI 3130×l genetic analyzer (Applied Biosystems). Peak height values obtained in probands were compared to those obtained in healthy controls, using GeneMarker 1.8 software (SoftGenetics, LLC, State College, PA).

### *RAI1* Sequence Analysis

Some patients were referred by their clinicians for commercial *RAI1* sequencing of exon 3 (GeneDx, Gaithersburg, MD) and enrolled in the NIH protocol with a confirmed *RAI1* mutation. DNA samples of these referred *RAI1* mutated patients, as well as DNA of our NIH contingent of other enrolled non-17p11.2 deleted SMS-like patients, were subsequently analyzed for all 4 *RAI1* coding exons, to accurately assess all gene variants (including SNPs). Primers were designed to amplify the 4 coding exons of *RAI1*, including their intronic boundaries in 22 amplicons (primer sequences available on request). Standard PCR amplification procedures were employed. All amplified products were directly sequenced using the BigDye 3 Terminator chemistry (Applied Biosystems) and separated on an ABI 3130×l genetic analyzer (Applied Biosystems). Data were evaluated using Sequencher 4.8 software (Gene Codes Corporation, Ann Arbor, MI).

### Missense Variant Prediction Tools

The effect of missense variations on protein function was evaluated using the mutation prediction programs POLYPHEN, PANTHER and PMUT.

#### POLYPHEN

(http://genetics.bwh.harvard.edu/pph/; POLYmorphism PHENotyping) predicts the effect of an amino acid substitution on the structure and function of a protein. POLYPHEN predictions are based on empirical rules that are applied to the sequence, as well as phylogenetic and known structural information that characterize the substitution. The Position-Specific Independent Counts (PSIC) is calculated for the two different alleles and the score for wild type and variant mapping to the known 3D structure [55].

#### PANTHER

(http://www.pantherdb.org/; Protein ANalysis THrough Evolutionary Relationships) estimates the likelihood of a non-synonymous variant to cause loss of function of the protein. The output, the subPSEC (substitution position-specific evolutionary conservation), is the negative logarithm of the probability ratio of the wild-type and mutant amino acids at a particular position based on a library. This library contains over 5,000 protein families and 30,000 subfamilies, each represented by a multiple sequence alignment and Hidden Markov Model. PANTHER subPSEC scores are continuous from 0 to −10. A value of 0 is interpreted as a functionally neutral variant; the more negative the subPSEC value, the more deleterious the substitution. The cutoff value suggested is −3 [56], [57], [58].

#### PMUT

(http://mmb2.pcb.ub.es:8080/PMut/) uses neural networks that have been trained with a large database of disease-associated and neutral variants to predict the impact of a given amino acid substitution. The output gives a neural network (NN) value between 0 and 1 (the higher this value, the more deleterious the variant) and a confidence value between 0 and 9 (the higher this value, the more reliable the NN) [59].

### *RAI1* mRNA Expression

Total RNA was isolated from patients' or control lymphoblastoid cells using the RNeasy Mini-Kit (Qiagen, Valencia, CA). RNA was subsequently treated with DNase (Applied Biosystems). RNA concentration and purity were assessed on a NanoDrop ND-1000 spectrophotometer (Thermo Fisher Scientific, Wilmington, DE). First strand cDNA was synthesized using a high capacity RNA-to-cDNA kit (Applied Biosystems). qPCR was performed utilizing two *RAI1* Assays-On-Demand Taqman primer-probe assays (Applied Biosystems), Hs00430773_m1 (Assay 1; located at the *RAI1* exon 2–3 boundary) and Hs01554690_m1 (Assay 2; located at the *RAI1* exon 3–4 boundary), and a control assay for the β-actin housekeeping gene (Hs99999903_m1). PCR amplifications were performed on 100 ng of cDNA using TaqMan Gene Expression Master Mix reagent (Applied Biosystems) and were carried out on an ABI PRISM 7900 HT Sequence Detection System (Applied Biosystems). Results were analyzed with the comparative Ct method as described [53], [60]. All assays were performed at least three times, and each sample was measured in triplicate. Displayed values in Figure 3 represent the relative quantification (RQ) normalized to the average of all control assays in all three control cell lines (arbitrary set to 1). For verification of results with an alternative control gene (to β-actin) with a similar threshold cycle (Ct) as *RAI1*, a *G6PC3* TaqMan assay (Hs00292720_m1) was used on selected mRNA samples (Figure S1). The average Ct for both *RAI1* assay 1 and *G6PC3* assays was ∼34–35 cycles in lymphoblastoid mRNA.

### Statistics

Data were compiled for statistical analysis using Statview. Differences between data groups were evaluated for significance using different standard statistical tests depending on the variables. For RNA expression data (Figure 3), where the number of patients/datapoints was not equal between the groups, the ANOVA post hoc Tukey-Kramer as well as the ANOVA Games-Howell tests were used. For phenotype-genotype correlations, specific tests (indicated in the text where used) included two-tailed unpaired t-test, non-parametric tests, Spearman's rank correlation coefficient (Spearman's rho). Chi-Square tests of independence were employed depending on whether the dependent variable was continuous or categorical. Given the concern for a potential outlier (M2543), the nonparametric Mann-Whitney test was used for means analysis of phenotypic features (Table 4). All data are presented as the mean ± SD (standard deviation). A *p*-value less than 0.05 was considered statistically significant.

## Supporting Information

Clinical Information S1 **Clinical description of** ***RAI1 de novo*** **variants,** ***RAI1 unclassified*** **variant,** ***RAI1 familial*** **variants.**
(DOC)

Figure S1 ***RAI1*** **mRNA expression in lymphoblastoid cells with different control assays.**
(DOC)

Figure S2 **Results of MLPA copy number analysis on genomic DNA from all lymphoblastoid cell lines used for** ***RAI1*** **expression analysis.**
(DOC)

Table S1 **(A)** ***RAI1*** **haplotype assignments to our non 17p11.2 deleted SMS patient cohort. (B) Haplotype analysis of alleles in A.**
(DOC)

Table S2 **Clinical data.**
(DOC)
