# Supplementary material for: Molecular Analysis of the Retinoic Acid Induced 1 Gene (RAI1) in Patients with Suspected Smith-Magenis Syndrome without the 17p11.2 Deletion
Source: PLoS One. 2011 Aug 8;6(8):e22861. doi: 10.1371/journal.pone.0022861 (PMC3152558; doi:10.1371/journal.pone.0022861)
Supplement: Clinical Information S1 — Clinical description of RAI1 de novo variants, RAI1 unclassified variant, RAI1 familial variants. (DOC) [file pone.0022861.s001.doc]

**Supporting Clinical Information S1**

**Clinical description of *RAI1* *de novo* variants:**

**Patient M2377** (previously reported as SMS159 [1]) was a 3.66kg, 53.3 cm male at birth, born vaginally at 42 weeks gestation to a 24-year-old G2P1 mother whose pregnancy was complicated only by flu-like illness at 6 months gestation. His early medical history^1^ documented significant infantile hypotonia with gross and fine motor delays but normal speech development. There was mild dilation of the lateral and 3^rd^ ventricles and spina bifida occulta (SBO); MRI at age 15 years identified Arnold-Chiari malformation, likely causative of his central vasovagal attacks. Video EEG for tonic clonic seizures was normal at age 5 years, with increased low frequency theta waves in the background of frontal lobe recordings. Genetic studies were initiated at age 13 years for suspected SMS due to craniofacial dysmorphia, obesity, gynecomastia (mastoplexy), hypogonadtropic hypogonadism, hoarse-deep voice, mild thoracolumbar scoliosis, mild intellectual disability and behavioral features highly suggestive of SMS that include “self-hugging”, aggressive and self-injurious behaviors (i.e., face slapping, polyembolokoilamania and onychotillomania). Fragile X and cytogenetic analysis, including FISH for 17p11.2, were normal. At age 19 years, an *RAI1* frameshift mutation c.1449delC [p.E484KfsX35] was identified [1].

NIH evaluation at age 20 years documented obesity (BMI 44.1 kg/m2) with truncal striae, weight of 152.3kg (>98^th^ centile), height of 185.8cm (91^st^ centile), and head circumference of 62cm (>95^th^ centile). Craniofacial features, consistent with SMS, included broad forehead, mild frontal bossing, flattened nasal bridge, malar hypoplasia, synophrys, youthful “cherubic appearance with rosy cheeks”, telecanthus, slightly cup-shaped ears, downturned “tented” upper lip, high-arched palate, prominent tongue papillae, and jaw deformity with open bite and relative prognathism. His gait was ataxic with normal reflexes. His voice was hoarse with rapid rate of speech and articulation abnormalities. Audiology testing identified unilateral mild high frequency sensorineural hearing loss (SNHL) for 4-6 kHz for the left ear. Distortion Product Otoacoustic Emissions (DPOE), 1-3 kHz in the left ear and 1-5 kHz in the right ear, suggested a cochlear contribution to his left SNHL. Laboratory studies were normal except for hypercholesterolemia and elevated prolactin and ACTH (Adrenocorticotropic hormone).

Neuropsychiatric and other testing were impeded by high levels of anxiety. Previous testing documented mild intellectual disability associated with DSM-IV co-morbidities/dual diagnosis (ADHD, ODD, anxiety disorder); academic function was at a 5^th^ grade level at age 18 years.

**Patient M2719** was a 3.4kg female born at 39 weeks gestation to a 25-year-old G2P1-2 mother after a normal pregnancy, labor and delivery. Initial genetic evaluation was pursued at 23 months of age due to central hypotonia, global developmental delay, dysmorphic facial features and a history significant for recurrent otitis media, GE reflux, obstructive sleep apnea with tonsillectomy and adenoidectomy (T&A) surgery, and febrile seizures. Genetic studies, including cytogenetic analysis for fragile X and FISH for Angelman/Prader-Willi syndrome and numerous metabolic studies, were all negative.

A suspected diagnosis of SMS prompted re-evaluation at age 13 years. Repeat cytogenetic analysis with FISH for the SMS critical region and subtelomeric probes and DNA-based fragile X studies were negative leading to pursuit of *RAI1* mutation analysis. A nonsense *RAI1* mutation was identified c.1973G>A [p.W658X]. The patient was a tall (81^st^ centile), obese (weight >98^th^ centile; BMI 34.3 kg/m2) female with mild intellectual disability, sleep disturbance, abnormal EEG behavioral issues, hypotonia, petit mal seizures with abnormal EEG (paroxysmal spike activity), mild/moderate conductive hearing loss, hyperopia, history of recurrent urinary tract infections and intermittent abdominal pain with gallstones (cholecystectomy at 11 years), L-sided kidney stone (at 15 years), and a grade II systolic murmur. Upper GI endoscopy performed at age 16 years for intermittent severe abdominal pain, nausea and vomiting was normal [2].

NIH admission at 17 years of age revealed a female with mild stigmata of SMS, global truncal obesity (BMI 38.6 kg/m2); mild bifid uvula without submucous cleft; normal hearing for speech/pure tones with slight air bone gaps consistent with childhood history of otitis media; mild thoracolumbar scoliosis (15 degree); small hands/feet with prominent fetal pads; and abnormal gait associated with leg length discrepancy (5/8 inch), pes planus, metatarsus abductus and recent complaints of plantar fascitis. Routine blood/serum chemistries were normal (Chem20 panel, CBC with differential, thyroid panel, fasting lipid panel, and quantitative immunoglobulins (IgA, IgG, IgM). Neurocognative assessment found impairments in adaptive function and intellectual disability in the mild range (FSIQ 68). She exhibits a behavioral phenotype consistent with SMS, i.e., emotional lability, anxiety, compulsive eating, aggressive/destructive outbursts, and self-injurious behaviors that include onychotillomania and polyembolokoilamania, but absence of self-hugging or hand-wringing behavior.

**Patient M2754** (previously reported as SMS335 [3]) was a 3.63kg female born at 42 weeks gestation to 30-year-old G2P1-2 mother treated with Macrodantin during pregnancy for urinary tract infections. Early infancy was notable for poor suck with feeding issues, generalized placid and socially remote demeanor with diminished cry, generalized hypotonia, and global developmental delays, especially in speech. Cytogenetic studies for suspected Prader-Willi syndrome (PWS) at age 30 months, due to persisting hypotonia and suspected eating disorder with weight gain, was negative; however, she was given a diagnosis of “atypical” PWS. Further genetic workup for global delays, mild facial coarsening and autistic-like features was pursued at age 8 years; DNA-based fragile X testing, PWS methylation assay, HGPRT enzyme activity for X-linked Lesch-Nyhan syndrome, genomic microarray, and numerous metabolic studies were all negative.

Parental suspicion for SMS led to referral at age 15 years that confirmed *RAI1* mutation c.3103dupC [p.Q1034PfsX31]. Features consistent with SMS include kyphoscoliosis identified at age 4 years, surgery for strabismus at 6 years, dental anomalies (missing the second premolars) and a profound right SNHL identified at 10 years. At diagnosis height was 14^th^ centile and weight >98^th^ centile (BMI 35.5 kg/m2, obese). Neurobehavioral findings included mild/borderline intellectual disability (FSIQ 62 at age 13 years) with dual diagnosis of **autism spectrum disorder (ASD) and pervasive developmental disorder (PDD)** at age 8 years. Chronic sleep disorder was present from early infancy, associated with temper tantrums during daytime sleepiness and fatigue, moderate obstructive sleep apnea and snoring, and a severe REM component by EEG-polysomnography (age 9 years).

NIH admission at age 19 years documented typical facial appearance of SMS with increased BMI (41.2 kg/m2); exophoria with myopia; mild truncal hypotonia with decreased muscle tone but good strength, slightly wide-based gait (pes planus); and scoliosis (30 degrees) without vertebral anomalies. Abdominal ultrasound revealed normal liver, spleen, and kidneys; echocardiogram was normal. Audiological testing confirmed right sensorineural hearing loss (1000-8000hz) with mild left loss (6000-8000hz). Routine blood/serum chemistries were normal for Chem-20 panel, thyroid panel, fasting lipid pane and quantitative immunoglobulins (IgA, IgG, IgM).

Her parents noted a shift in behavior after age 3 years that, by school age, was characterized by aggressive outbursts, increased obsessions about food, poor impulse control and rapid mood swings, attention seeking, and self-injurious behaviors (skin picking, onychotillomania), but notable absence of self-hugging. At 19 years of age, her most persistent behaviors are excessive eating/food seeking, and severe skin picking (provoked by anxiety and boredom, but probably not to engage attention) for which she takes Prozac without significant improvement.

**Patient M2911** was a 3.18kg female born by C-section at 37 weeks gestation to G2P1-2 mother after an uncomplicated pregnancy. Past medical history was significant for mild neonatal jaundice, severe gastroesophageal reflux disease (GERD) that resolved at about 19 months of age, surgery for congenital dacryostenosis, middle ear dysfunction with frequent ear infections requiring repeat pressure equalizing (PE) tube placement between ages 12-16 months, mild-moderate conductive hearing loss, allergies (at 19 months), pneumonia (at 20 months), mild hypotonia with secondary weakness, joint laxity (evidenced by genu recurvatum, hyperextensible fingers, and planovalgus ankles), without dislocations, labial adhesions (at 20 months), and a voracious appetite with obesity (BMI 24.5 kg/m2; weight >98^th^ centile; height 27^th^ centile) at age 5 years. Speech evaluation at approximately age 20-22 months documented significant delays in speech (expressive language more delayed than receptive language), language and oral motor development with diminished babbling/speech sounds, unusual pattern of word acquisition, and significant oral-motor dysfunction (lax open mouth posture with protruding tongue, excessive drooling, and mouthing objects). Gross and fine motor skills assessed at 18 months of age were delayed to approximately 10-13 month level. Initial psychiatric evaluation at age 4 years led to dual diagnosis of autistic disorder/Asperger-like, ADHD and behavior disorder. At 4 years expressive language remained delayed, but receptive skills were in the normal range; FSIQ measured 62 (1^st^ centile) by Wechsler Nonverbal Scale of Abilities/ Behaviors included a distinct pattern of rigidity about daily routines and rituals, rapid mood shifts with daily tantrums, ADHD, significant impulsivity, low attention span, easy distractibility, and difficulties reading non-verbal cues or other’s emotions. She exhibited an insatiable appetite, possibly further exacerbated by medication(s). The family history was significant for anxiety issues for several maternal relatives. Psychotropic medication trials targeting behavior showed some initial benefit with risperidone and Abilify, but effects waned over time and adverse side effects were noted.

Cytogenetic analysis with FISH for SMS, DNA methylation analysis for PWS, DNA-based fragile X testing, oligoarray-CGH, and metabolic studies were all normal. At age 5 years Kaiser Parmanente Genetics referred her for *RAI1* mutation analysis, resulting in identification of a frame-shift *RAI1* mutation c.548delT [p.L183RfsX69].

**Clinical description of *RAI1* *unclassified* variant:**

**Patient M2543** was a 2.95kg male born at 38 weeks gestation to a 24-year-old G2P1-2 Indian mother and European father after normal pregnancy; the neonatal period was uncomplicated. At referral (age 14 years) he exhibited moderate developmental delay/intellectual disability with dual diagnosis of ADHD/ASD, chronic sleep disorder (nocturnal awakenings with daytime naps), marked behavioral problems consistent with SMS and craniofacial features of SMS (i.e., brachycephaly, mid-face hypoplasia, slightly coarse appearance with full heavy eyebrows, high arched palate, and overbite with crowded teeth (prolonged retention)), normal head circumference (10^th^ centile), short stature with decreased subcutaneous fat. Early childhood was significant for swallowing and feeding issues, frequent ear infections, left esotropia, mild hand tremor and broad based gait with decreased exercise tolerance. At 17.6 years height and weight remained at the ≤2^nd^ centile with head circumference at 2^nd^ centile. BMI was in the normal range. Additional findings included hoarse voice, seizures until age 5 years, brachydactyly, daytime enuresis to 5 years, and moderate intellectual disability. Behavioral abnormalities include self-hugging, anxiety, rapid mood shifts (“Jeckyl & Hyde”), aggressive outbursts, self-injurious behaviors (i.e., headbanging, self hitting and biting, skin picking, nail biting), property destruction (punched holes in walls), mouthing and chewing of objects, compulsive eating/food foraging with pica, and, as younger child, poop-smearing. Sleep remains a major issue, with major settling issues and decreased total nighttime sleep for age.

Prior genetic studies, including cytogenetic analysis with FISH for the 17p11.2 SMS deletion, fragile X testing, and numerous metabolic studies, were all negative. *RAI* mutation testing was pursued at age 14 years, identifying a missense c.725C>T [p.P242L] and a silent c.2907C>T [p.D969D] variant in *RAI1*. The missense variant was not present in either his clinically unaffected mother or brother; the father was unavailable for testing.

**Clinical description of *RAI1* *familial* variants:**

**Patient M2365** was a 4.5kg, large-for-gestational age (length >95^th^ centile), white male born by emergency C-section for cephalopelvic disproportion at 39 weeks gestation to a 28-year-old G5P2-3-SAB2 mother. Neonatal issues included jaundice, mild heart murmur, hypotonia and poor suck and swallow. He was hospitalized numerous times for infections after age 6 months, including for pneumonia, recurrent sinusitis, otitis media, severe gastroenteritis (rota virus), bowel infections (suspected candida), scarlet fever, and parainfluenza. Due to chronic otitis media and mastoid infections he required repeated radical mastoidectomies of the left ear (at 18 months, 2, 6.5, and 7.5 years). This led to a diagnosis immunodeficiency (IgG subtype abnormality) and initiation of monthly IVIG therapy at 7.5 years. Iron therapy was initiated at 18 months of age for iron deficiency anemia due to repeated epistaxes. Genetic evaluation at 9 years was suggestive of SMS, including early global developmental delays and mild intellectual disability range of function (FSIQ 62), dual diagnosis of ASD and ADHD, height (21^st^ centile) and weight at 59^th^ centile with “overweight” BMI (17.9 kg/m2), dysmorphic facies (broad square shaped face, midface hypoplasia, hypertelorism, synophrys, relative prognathism, “tented” upper lip, low set ears), hoarse/raspy voice, left-sided hearing loss, small hands/feet (brachydactyly) with single palmar crease, hand tremor, broad-based flapping gait, hypotonia with muscle weakness, immune deficiency, and diagnosed seizure disorder. Currently at age 16.5 years he has delayed puberty (Tanner 1) and delayed bone age (13.5 years) with suspected hypothalamic dysfunction, continued epistaxes, and symptoms of Raynaud’s (cold hands that turn blue). Behaviors suggestive of SMS included affectionate, endearing personality with variety of self-injurious and maladaptive behaviors, i.e., aggressive outbursts, temper tantrums, anxiety, impulsivity, hyperactivity, major mood swings, property destruction (walls, windows), head banging, nail pulling, skin picking, and self-hitting).

Prior genetic studies included normal cytogenetic analysis (46,XY; 550-575 band resolution) with normal FISH for SMS including both commercial (Cytocell) and *RAI1*-specific (RPC-253p7) probes, normal mitochondrial mutation analysis and normal parental karyotypes. *RAI1* mutation analysis was pursued at 9 years of age and identified a missense variant c.5653G>A [p.D1885N] as well as a silent variant c.3183G>A [p.T1061T], which were both identified in his unaffected father, and were absent from his mother’s DNA.

His father, now 45 years of age, is a regular long-distance runner with height 182 cm (80^th^ centile), weight 97kg (85^th^ centile) and BMI 29.3 kg/m2 (overweight for age). He has normal intelligence, behavior, vision and hearing, and is non-dysmorphic. His past history was significant for childhood infections that included rheumatic fever & CMV, recurrent sinusitis, and complaints of leg cramps and sports-related injuries.

**Patient M2732** was a 2.81kg AGA white female born at 37 weeks gestation to a 32-year-old G4P2-3SAB1 mother with a history of depression treated for 1 year prior to pregnancy. Prenatal history was uncomplicated except for maternal smoking (3-5 cigarettes/day) and Zoloft 25 mg taken daily in the last 3 months of pregnancy. The neonatal period included a 3 week admission to the newborn intensive care unit for tachypnea with intermittent stridor attributed to mild laryngomalacia, hypotonia, failure to thrive with nasogastric tube feeds, mild jaundice treated with phototherapy, symptoms of withdrawal due to maternal Zoloft use, and mild dysmorphic features. Cytogenetic analysis (550-band resolution) after birth included FISH and was negative for telomeres and 22q deletion [46,XX.ish(subtelx2; .ish22q11.2(D22S75x2)]. Newborn metabolic and hearing screens were normal and DNA-testing for myotonic dystrophy (myotonin protein kinase; *DMPK* gene) pursued due to a positive family history was also negative. Brain MRI confirmed subdural hematoma secondary to delivery and an old choroid plexus hemorrhage without hydrocephalus, mild irregular narrowing of the right transverse sinus with mild right nasolacrimal duct obstruction. Her early childhood was significant for recurrent middle ear dysfunction, mild gastroesophogeal reflux (GERD) with vomiting, mild laryngomalacia; pseudo-strabismus with moderate hyperopia (7 months), normal EEG (at 13 months), and referral for speech, occupational and physical therapy services.

At age 16 months, a further genetic workup was pursued for suspected SMS due to dysmorphic facial features, global developmental delay, hypotonia, episodes of breath-holding and crying, squeezing herself (“self-hug”), significant oral-motor dysfunction and recurrent otitis media with pressure-equalization (PE) tubes. Normal CGH-array (1887 BAC clones; Signature™ chip) results led to a referral for *RAI1* mutation analysis that identified a novel missense variant c.707A>T [p.Y236F]. Parental studies identified the same p.Y236F change in the mother, whose history is significant for her father, two aunts and an uncle with myotonic dystrophy and onset of cataracts in their 30-40’s. She had cataract removal at age 29 years of age and is otherwise healthy, except for treatment of depressive symptoms.

Additional SMS-like features presented at her NIH exam at age 27 months included height at 26^th^ centile, weight at the 20^th^ centile with “obese” BMI (16.0 kg/m2), head circumference at 75^th^ centile, central hypotonia with global motor and speech delays, dysmorphic facial appearance (upturned palpebral fissures, blue “stellate” irides, broad flat nasal bridge with telecanthus/pseudostrabismus), rosy cheeks, short philtrum, down-turned “tented” upper lip, anterior deep over-bite, mild micrognathia, high arched palate, speech delay (non-vocal with use of 10 signs) with oral-motor dysfunction and lingual papillae, small umbilical hernia, hyperextensibility of her fingers, persistent fetal pads on fingers and toes, a broad based unsteady gait, occasional constipation, mild gastroesophageal reflux treated with Zantac for 1 year, history of sleep disturbance (increased daytime naps, nocturnal awakenings) with EEG-polysomnography documented obstructive sleep apnea at 20 months, stereotypies and self-injurious behaviors (“self-hug”, head-banging, skin picking; temper tantrums) and normal echocardiogram.

**Patient M2826** was a 1.89kg 34-week white female born prematurely to a prima gravida 19- year-old mother. The pregnancy was complicated by maternal binge drinking limited to the early weeks before pregnancy was confirmed and ultrasound monitoring for intrauterine growth retardation. She remained in neonatal ICU for 2 weeks due to prematurity, jaundice requiring 4 days phototherapy, and temperature instability. Her medical history is significant for generalized lethargy in infancy with poor suck/swallow, exaggerated gag reflex until age 4 years, global delays, mild dysmorphic features, allergies and multiple ear infections, unilateral left mild-moderate and right mild low frequency hearing loss documented at age 2 years, frequent upper respiratory infections (chronic sinusitis, recurrent pneumonia to age 5 years), croup, scarlet fever and whooping cough despite immunization, generalized hirsutism present since birth, partial complex seizure disorder (absence seizures), urinary incontinence, dental malocclusion, probable left ventricular hypertrophy on electrocardiogram (6 years), and hypertension felt secondary to Dexadrine. Psychological assessment at age 8 years documented borderline cognitive function with significant academic deficits, severe deficits in language and adaptive functioning and visual-motor integration problems. Several syndromes initially considered but ruled included alcohol spectrum disorder, Cornelia de Lange, William syndrome, and neurofibromatosis (3 café au lait spots; negative Wood’s lamp exam), and McCune-Albright (normal skeletal survey). CF-sweat test and metabolic studies (plasma and urine amino acids, MPS screen; plasma homocysteine) were normal, except for mild elevation of plasma glutamine; follow-up serum ammonia was normal (14; normal range 12-47 umol/L). An elevated platelet count was documented at age 5 years (721K; normal range 150K-400K per mL). At age 9 years, a workup for lipodystrophy was pursued due to hypertrichosis, increased neck pigmentation and decreased adipose tissue. Normal imaging studies included head CT, and abdominal/renal ultrasounds (9 years), At referral at age 10 years, she was normocephalic (25^th^ centile) with height at 13^th^ centile and weight at 21^st^ centile yielding normal BMI (16.3 kg/m2).

Further NIH admission at age 12.5 years documented normal BMI (19.2 kg/m2), mild dysmorphic facial appearance with bulbous nasal tip, anteverted nares, upslanting palpebral fissures; dental malocclusion with caries; mild swallow delay and reflux by swallow study; hyperopic astigmatism; hypotonia, mild hypertrichosis, mild scoliosis and small joint hyper extensibility of fingers with persistent fetal pads and bilaterally short 5^th^ fingers. Routine blood/serum chemistries, including CBC with differential, glucose, insulin, liver function tests, thyroid function, fasting lipid panel, quantitative immunoglobulins were normal. Echocardiogram, brain MRI and abdominal ultrasound were normal. Medications included Dexadrine-SR and Strattera in the morning, and clonidine and melatonin at bedtime. Neurocognitive assessment documented impaired cognitive function (borderline) with Abbreviated Battery IQ of 76 (Stanford Binet) and significant impairment in all areas of adaptive function. Neurobehavioral findings were consistent with SMS and included dual diagnosis of ADHD, sensory issues, decreased pain sensitivity, sleep disturbance with settling difficulties, nocturnal awakening, and suspected sleep apnea, recent weight loss despite voracious appetite and food-seeking behavior, and severe behavioral disturbances with outbursts, impulsivity, rapid mood swings and self-injurious behaviors.

Prior genetic studies, including cytogenetic analysis (550 band resolution) with telomere FISH, fragile X testing (normal CGG repeat range) were normal. At age 10 years mutation testing for *RAI1* was pursued, identifying a heterozygous novel missense variant c.3208G>A [p.G1070R] as well as a novel silent variant c.4512G>T [p.L1504L] that were both also present in her mother.

The mother of M2826, age 32 years, is tall (>95^th^ centile) and experienced a significant weight gain in past few years (high of 200 pounds) with current weight of 78.9kg (92^nd^ centile) and “overweight” (BMI 26 kg/m2). She is cognitively normal, but had early speech delays with speech impediment and learning problems in school (visual learner), received speech therapy from age 6-12 years, and program supports with modification during her school years. Her medical history is significant for cleft palate, overbite, vision problems with astigmatism and right eye “blindness” due to amblyopia that did not respond to patching in childhood, recurrent upper respiratory infections from childhood to present, mild bilateral hearing loss with history of recurrent ear infections as a child and frequent mastoiditis of left ear in adulthood, allergies (bees and seafood), cold hands/feet that turn blue due to poor circulation (possibly Raynaud’s), and recent diagnosis of rheumatoid arthritis with vitamin D deficiency. She reports being a very “moody and defiant” child, but did not have any major behaviors and few sleep issues until adolescence when she could sleep all day if given the opportunity. The biologic father of M2826, who was unavailable for study, is reported to have family history significant for developmental delay/intellectual disability, attention deficits and mental health issues.

**Patient M2867** was a 4.139 kg (>90^th^ centile) large for gestational age (LGA) white female delivered by C-section at 38 weeks gestation to a 38-year-old G3P1-2AB1 mother. Prenatal history was significant for amniocentesis for advanced maternal age that revealed a normal 46,XX fetal karyotype. Neonatal history was significant for fractured right clavicle and plagiocephaly noted at delivery, infantile hypotonia, irritability with frequent emesis leading to GERD diagnosis (age 6 months), feeding problems with mild-moderate dysphagia and excessive drooling that persisted into childhood, and diminished sleep for age. Additional medical history included multifocal seizures with abnormal EEG (increased spike activity) treated with anticonvulsants from age 7 months, psychomotor delays recognized at age 8.75 months (i.e., cognitive, receptive and expressive language skills at 5 month level, gross and fine motor skills at 3.75 and 2.75 month levels, respectively), normal endocrine workup for suspected precocious puberty (downy pubic hair) at 9 months, history of frequent infections (pneumonia, chronic otitis media, upper respiratory infections) and H-flu influenza despite earlier vaccination suggesting immune disorder, suspected Raynaud’s (4 years) with tilt-table workup for autonomic dysfunction (dysautonomia) suggesting possible mild sympathetic cardiovascular and vasomotor over activity, chronic constipation, fecal and urinary incontinence at age 7 years, and normal eye exam, except for pale optic nerve. Repeat MRI studies documented ventriculomegaly and evidence of delayed myelination at age 3 years when compared to age 1 year.

Genetic referral and NIH evaluation at 5.3 years revealed a tall (>98^th^ centile), lean appearing female with reduced BMI 13.7 kg/m2 (normal). Head circumference, which was 50^th^ centile at birth, measured 25^th^ centile at 5 years. She was non-ambulatory with global developmental delay. Prior developmental assessment at 33 months documented cognitive skills at 13 months, gross and fine motor skills at 10 months, and language/communication at 10 months and sensory issues.

Features consistent with SMS included generalized hypotonia, fair-complexion with rosy cheeks, hypertelorism, prominent eyebrows with synophrys, long eyelashes, relative prognathism, dry skin, myopia (wears glasses), and persistent fetal pads on fingers 3-5, Tanner I with downy dark pubic hair (premature adrenarche). She also exhibited mild hirsutism and long fingers and toes. Developmentally she was globally delayed in all areas, but has continued to make progress without evidence of developmental regression. She walked with a broad based gait with support/walker; speech was limited to signs with attempts to mimic words/sounds and several spoken words. Behavioral features included general happy demeanor, with chronic bruxism (requiring dental capping), page flipping, insertion of hands/objects in mouth but no evidence of aggressive outbursts, tantrums, “self-hugging” or self-injurious behaviors. While sleep disturbance was noted at younger ages, parental report suggested a pattern of nocturnal awakenings lasting ~1 hour for several months, shifting to uninterrupted sleep (without major awakenings) for several months; no formal sleep study was performed.

Prior genetic studies included repeated cytogenetic analysis (46,XX) with FISH for telomeres and for SMS, methylation studies for PWS/AS, CGH array (1887 BAC clones; Signature™ chip deletion/duplication), *MECP2, UBE3A, RPS6K3* and *CKL5* genetic analysis, and extensive metabolic workup (amino acids, organic acids, 7-dehydrocholesterol, CDG-transferrin), all negative. *RAI1* mutation analysis identified the novel c.3781_3783delGAG [p.del1261E] variant that was also found in the father. The father’s history is significant for pediatric endocrine evaluation for gynecomastia at age 7 years; asthma, joint problems (arthritis of foot, bursitis of elbow and complaints of back pain); sinusitis & ENT issues with unilateral hearing loss (felt due to loud music); hypertension, and antidepressant treatment due to familial depression/mental health problems.

**Patient M2900** was a 2.9kg (50^th^ centile) Hispanic male with a 2-vessel cord prematurely born at 33.4 weeks gestation to a 28-year-old G4P3-4 mother from the Dominican Republic. His medical history was significant for 6 week hospitalization for prematurity and neonatal apnea, distinct physical features, macrosomia (height, weight and head circumference >98^th^ centile) with normal bone age, issues of allergies/eczema and moderate persistent asthma requiring hospitalization, ongoing sleep disturbance (nocturnal awakenings, daytime lethargy) despite tonsillectomy and adenoidectomy (T&A) at age 3 years for obstructive sleep apnea, epistaxis post T&A, frequent ear infections, sinusitis and upper respiratory infections, atopic dermatitis (at 5 years), delayed eruption of secondary teeth, functional Still’s murmur, abnormal EEG with multiple bi-occipital (R>L) spike wave complexes with broad field potentiated by sleep (at 5.5 years), developmental/cognitive delays with significant speech/language impairment (expressive/receptive), autistic-like features, i.e., strengths with rote skills but lacking functional play, language or social interaction but did not meet criteria for PDD-NOS (Pervasive Developmental Disorder, Not Otherwise Specified), and behavioral difficulties that include stereotypies (hand-flapping; twirling self), headbanging, nail-biting, mouthing non-food objects/pica, hyperphagia, perseveration on topics of interest (e.g., obsessions about signs, labeling things and toilets); and significant oppositionality and aggression.

Extensive prior genetic workup was negative, including, cytogenetic analysis (46, XY; 550 band resolution); CGH-array, fragile X (*FMR1* sequencing and DNA methylation analysis); mutation analysis for autism panel (*NLGN3, NLGN4, STK9/CDKL5*), MRX-panel (X-linked genes *ARX, DLG3, FACL4, FTSJ1, JARID1c, PQBP1, TM4SF2*, and *ZNF41*); Rett syndrome (*MECP2* sequence and deletion analysis); FG syndrome (*MED12* gene exons 4,5, 21,22, 28 or 36); Soto syndrome (*NSD1* gene); and Bannayan-Ruvalcaba-Riley/Cowden syndrome (*PTEN* gene copy number and sequencing); congenital disorders of glycosylation (transferrin); and metabolic studies (monoamine oxidase A (MAO-A), amino and organic acids). Routine lab chemistries and urinalysis at age 8 years were essentially normal, including lead level, thyroid function and lipid panel.

Genetic referral and NIH evaluation at almost 6 years with a deletion negative SMS-like phenotype. He was tall (>98^th^ centile), obese (weight >98^th^ centile; BMI 23.7 kg/m2), with a deletion negative SMS-like phenotype that included craniofacial dysmorphology (i.e., broad square shaped face, deep-set eyes with upslanting palpebral fissures, arched/heavy eyebrows, mild hypertelorism, maxillary hypoplasia, tented upper lip, and mild micrognathia), hoarse voice, brachydactyly with persistent fetal pads, history of constipation and enuresis, developmental/speech delay, and sleep and behavioral issues. Renal ultrasound was normal. *RAI1* sequence analysis of patient **M2900** identified heterozygosity for the novel silent *RAI1* variant c.1500G>A [P500P] and rare *RAI1* variant rs61746214: c.3791G>A [p.E1264G]. These same variants were present heterozygous in his developmentally delayed dysmorphic 8-year-old brother (**M2901**) described below and a cognitively normal non-dysmorphic 4.5-year-old sister (**M2902**) who, except for single urinary tract infection (age 1 year), has no major medical issues. The mother of M2900 (**M2903**) was homozygous for c.1500G>A [P500P] and heterozygous for rs61746214: c.3791G>A [p.E1264G]. The father’s DNA was not available for testing.

The 8-year-old brother (**M2901**) had global developmental delays (both motor and speech), autism spectrum disorder (ASD), and similar dysmorphic features as well as a similar tall, obese body habitus (BMI) to his affected brother (**M2900**). **M2901** was a 3.2kg term Hispanic male at birth, with past medical history significant for neonatal jaundice, lower lid chalazion; intermittent vomiting at age 1 year with negative upper GI series; developmental delay/moderate intellectual disability, repetitive behaviors and language impairment that meets criteria for ASD; obstructive sleep disturbance that resolved post T&A; and without vision, spine, cardiovascular, or genitourinary abnormalities. Initial genetic evaluation at age 6.5 years documented a tall, obese body habitus with head circumference 54.5cm (98^th^ centile) and height and weight >98^th^ centile (BMI 19.1 kg/m2), craniofacial features that included dolichocephalicy, broad nasal root, deep-set eyes with hypertelorism, and over-folded helices. Other features included mild pectus, bilateral 5^th^ finger clinodactyly, broad thumbs and great toes, persistent fetal pads (right hand 15cm; palm 8.75cm) and hyperconvex nails. Prior genetic studies included normal 46,XY karyotype, fragile X testing, and metabolic studies (plasma amino acids, urine organic acids).

The mother (**M2903**), a 35-year-old cognitively normal Hispanic G5P4-SAB1 female was 165cm (60^th^ centile) tall. Her exam and history were significant for dysmorphic facial features (round face; broad nose) distinct from her family and suggestive of SMS, history of sleep disturbance since early childhood with difficulty staying asleep at night, and suspected early speech delays (she did not speak in sentences until age 4 years). Behaviorally she reports no aggressive behavior or temper tantrums, but admits to irritability, sudden mood changes, and a low frustration tolerance. After the birth of **M2900**, she was documented to have a profound left-sided hearing loss and normal renal ultrasound. Her family history was negative for relatives with behavioral or sleep issues.

**References**

1.Slager RE, Newton TL, Vlangos CN, Finucane B, Elsea SH (2003) Mutations in RAI1 associated with Smith-Magenis syndrome. Nat Genet 33: 466-468.

2. Chen E, Levy H (2007). Additional genotype-phenotype correlations in a patient with Smith-Magenis who as a novel point mutation in the RAI1 gene. Am. College Med. Genetics 2007 Annual Meeting, Abstract 158. Available at http://submissions.miracd.com/acmg/.

3. Truong HT, Dudding T, Blanchard CL, Elsea SH (2010) Frameshift mutation hotspot identified in Smith-Magenis syndrome: case report and review of literature. BMC Med Genet 11: 142.
