# Supplementary material for: Molecular Analysis of the Retinoic Acid Induced 1 Gene (RAI1) in Patients with Suspected Smith-Magenis Syndrome without the 17p11.2 Deletion
Source: PLoS One. 2011 Aug 8;6(8):e22861. doi: 10.1371/journal.pone.0022861 (PMC3152558; doi:10.1371/journal.pone.0022861)
Supplement: Figure S1 — RAI1 mRNA expression in lymphoblastoid cells with different control assays. (DOC) [file pone.0022861.s002.doc]

**Supporting Figure S1**

**
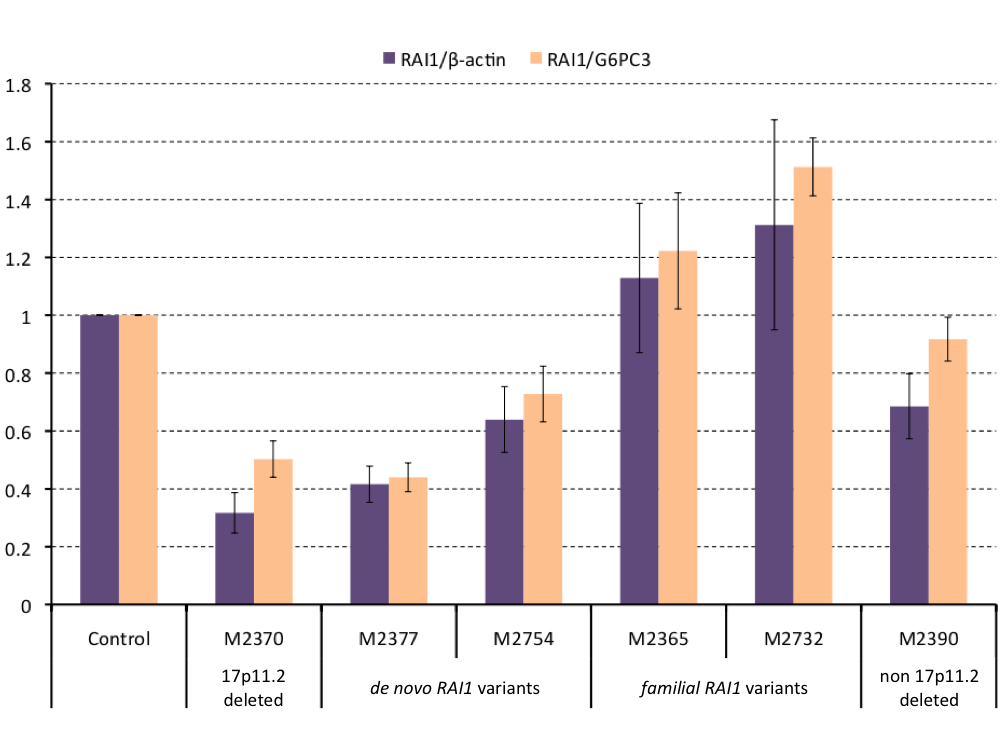
**

**Figure S1. *RAI1* mRNA expression in lymphoblastoid cells with different control assays**

qPCR results of RNA extracts from lymphoblastoid cells from selected SMS patients from the 4 subgroups displayed in Figure 3 (main text). The Taqman *RAI1* assay #1 was used and results were normalized to the *G6PC3* Taqman assay. The *RAI1* and *G6PC3* assays displayed a similar threshold cycle (Ct) of ~34-35 cycles in lymphoblastoid cells. All experiments were performed in triplicate, with three replicates per experiment. Displayed values represent the relative quantification (RQ) compared to the average of the control assays (set to 1). Results with *G6PC3* as normalizing gene are displayed in purple, and compared to the original results in orange with beta-actin (Ct of ~ 26-27 cycles) as normalizing gene (as displayed in Figure 3, main text). The average *RAI1* mRNA expression levels are in the same range (slightly increased, mostly within the error margin) with *G6PC3* compared to ß-actin as normalizing gene.
