# Supplementary material for: Molecular Analysis of the Retinoic Acid Induced 1 Gene (RAI1) in Patients with Suspected Smith-Magenis Syndrome without the 17p11.2 Deletion
Source: PLoS One. 2011 Aug 8;6(8):e22861. doi: 10.1371/journal.pone.0022861 (PMC3152558; doi:10.1371/journal.pone.0022861)
Supplement: Figure S2 — Results of MLPA copy number analysis on genomic DNA from all lymphoblastoid cell lines used for RAI1 expression analysis. (DOC) [file pone.0022861.s003.doc]

**Supporting Figure S2**


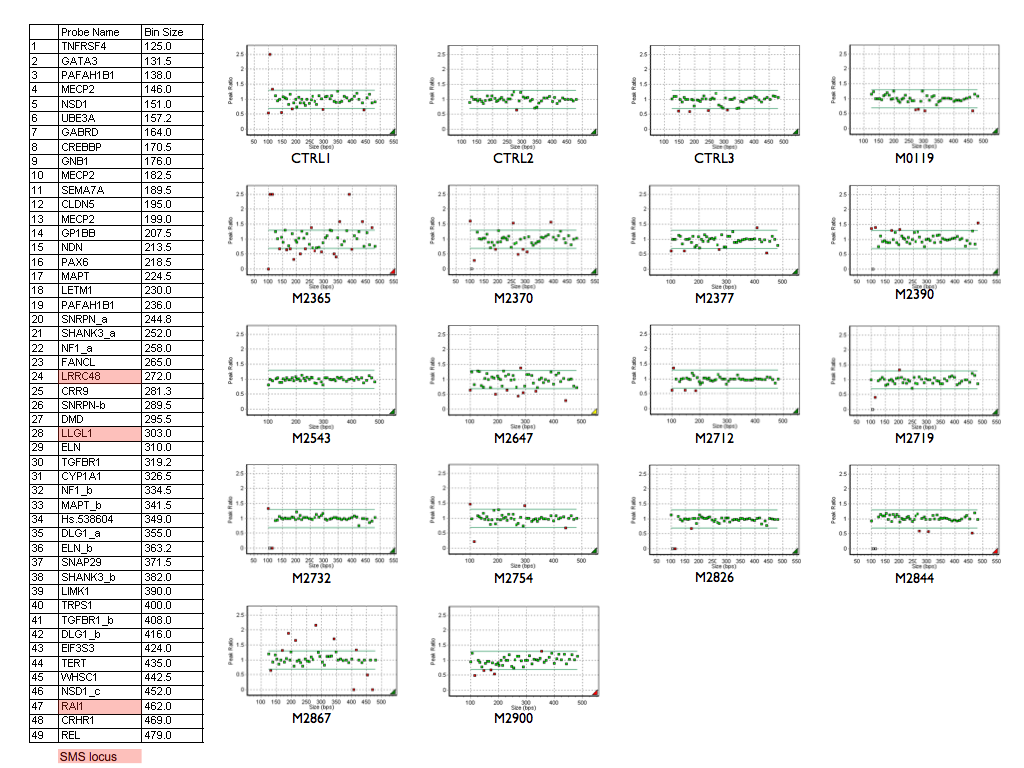


**Figure S2. Results of MLPA copy number analysis on genomic DNA from all lymphoblastoid cell lines used for *RAI1* expression analysis**.

Since genomic copy number variations are a concern when using lymphoblastoid (EBV transformed) cells, multiplex ligation-dependent probe amplification (MLPA) analysis was performed on genomic DNA from all cell lines that were used for *RAI1* mRNA expression studies. The MLPA P245-A2 Microdeletion Syndromes-1 kit (MRC-Holland, Amsterdam, The Netherlands) was used for this analysis, containing 49 genes (listed in the Table on the left) including 3 genes in the 17p11.2 SMS critical region: *RAI1*, *LLGL1*, and *LRRC48* (highlighted). The data were analyzed with GeneMarker 1.8 software (SoftGenetics, LLC, State College, PA). The ratio plots on the right show the relative signal intensities of all probes plotted according to the gene order listed in the table. All tested cell lines were confirmed to have two alleles for *RAI1*, except for the 17p11.2 deleted cases (M2370, M0119, M2844), who were confirmed to have one copy for the 17p11.2 genes *RAI1*, *LRRC48*, and *LLGL1*. Cell lines M2365, M2370 and M2867 showed a variety of abnormal copy number variations outside the 17p22.1 region, which need to be taken into account for future analysis of these cell lines or these patients.
