# Supplementary material for: Molecular Analysis of the Retinoic Acid Induced 1 Gene (RAI1) in Patients with Suspected Smith-Magenis Syndrome without the 17p11.2 Deletion
Source: PLoS One. 2011 Aug 8;6(8):e22861. doi: 10.1371/journal.pone.0022861 (PMC3152558; doi:10.1371/journal.pone.0022861)
Supplement: Table S1 — (A) RAI1 haplotype assignments to our non 17p11.2 deleted SMS patient cohort. (B) Haplotype analysis of alleles in A. (DOC) [file pone.0022861.s004.doc]

**SUPPORTING TABLE S1:**

**TableS1A. *RAI1* haplotype assignments to our non 17p11.2 deleted SMS patient cohort ^a^**


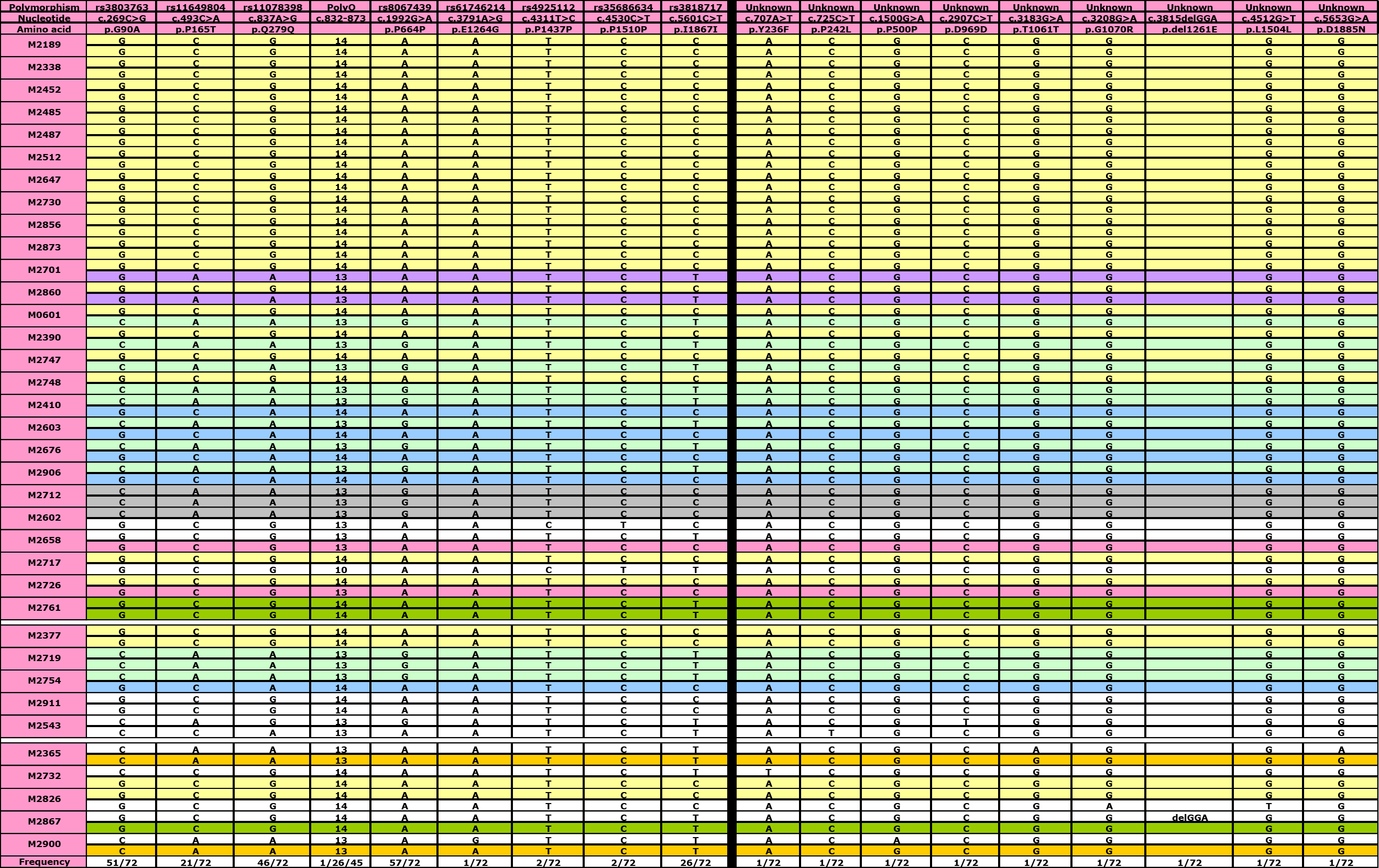


^a^ DNA variant numbering system is based on *RAI1* cDNA (NM_030665; with nucleotide +1 corresponding to the A of the ATG initiation codon). Known SNPs (indicated by their *rs* accession number of dbSNP: <http://www.ncbi.nlm.nih.gov/snp/>) are indicated on the left of the table. Unreported (familial) variants are indicated on the right.

| **Table S1B. Haplotype analysis of alleles in Table S1A^a^** | | |
| --- | --- | --- |
| *Haplotype* | *# of*  *alleles* | *Allele*  *frequency* |
| **H1** | **32** | **44 %** |
| **H2** | **11** | **15 %** |
| **H3** | **5** | **7 %** |
| **H4** | **2** | **3 %** |
| **H5** | **3** | **4 %** |
| **H6** | **2** | **3 %** |
| **H7** | **3** | **4 %** |
| **H8** | **2** | **3 %** |
| **u (unique)** | **12** | **17 %** |
|  | **72** | **100 %** |

**^a^** For most patients the listed haplotypes are the only possible combination of variants; for others the haplotype is the most likely prediction. We prioritized the presence of a ‘common haplotype’ allele (Haplotype 1 (H1) in yellow), and then assigned the nucleotides of the second allele. Eight more frequently occurring haplotypes were identified (H1-8), as well as several unique haplotypes (u, white background). Allele frequencies for each haplotype are indicated.
