# Supplementary material for: Molecular Analysis of the Retinoic Acid Induced 1 Gene (RAI1) in Patients with Suspected Smith-Magenis Syndrome without the 17p11.2 Deletion
Source: PLoS One. 2011 Aug 8;6(8):e22861. doi: 10.1371/journal.pone.0022861 (PMC3152558; doi:10.1371/journal.pone.0022861)
Supplement: Table S2 — Clinical data. (DOC) [file pone.0022861.s005.doc]

| **Supporting Table S2. Clinical data** | | | | | |  |  | | | | |  |  | | | |
| --- | --- | --- | --- | --- | --- | --- | --- | --- | --- | --- | --- | --- | --- | --- | --- | --- |
|  | ***de novo* cases** | | | | |  | ***familial* cases** | | | | |  | **Summary** | | | |
| **Patient #** | **M2377** | **M2719** | **M2754** | **M2911** | **M2543^a^** |  | **M2365** | **M2732** | **M2826** | **M2867** | **M2900** |  | ***de novo***  **(n=5)** | **del 17p11.2** | ***RAI1* mutation** | |
| ***RAI1*** |  | | | | |  |  | | | | |  | **Current study** | **Previous Reports [1,2]** | | |
| Variant | c.1449delC p.E484Kfs | c.1973G>A p.W658X | c.3103insC p.Q1034Pfs | c.548delT p.L183Rfs | c.725C>T p.P242L |  | c.5653G>A p.D1884N | c.707A>T p.Y236F | c.3208G>A p.G1070R | c.3781_3783 delGAG | c.1500G>A p.P500P |  |  |  | |  |
| Parental origin |  |  |  |  |  |  | Paternal | Maternal | Maternal | Paternal | Maternal |  |  |  | |  |
| mRNA level % (SD) | 35.9% (± 8.3) | 58.7% (± 25.3) | 54.8% (± 13.4) | NA | 59.7% (± 19.6) |  | 98% (± 28.0) | 105% (± 38.1) | 47% (± 18.6) | 20.7% (± 4.8) | 60.9% (± 17.4) |  | 52.3%  (± 19.3) |  | |  |
| Haplotype | H1/H1 | H2/H2 | H2/H3 | u/u | u/u |  | H8/u | H1/u | H1/u | H5/u | H8/u |  |  | |  |
| **Growth** |  | | | | |  |  | | | | |  |  | | | |
| Gender, Age | M, 20y | F, 13y | F, 15y | F, 5y | M, 17.6y |  | M, 9y | F, 27mo | F, 10y | F, 5.3y | M, 6y |  | 3F/2M |  | |  |
| Gestational age  (weight centile) | 42 wk AGA (50%) | 39 wk AGA (50%) | 42 wk AGA (50%) | 37 wk AGA (50%) | 38 wk AGA (10%) |  | 39 wk LGA (>95%) | 37 wk AGA (50%) | 34 wk IUGR (25%) | 38 wk LGA (>90%) | 33.4 wk AGA (50%) |  | $\bar{x}$=39.6wk  ± 2.2 | 80% term **[3]** | | term  AGA^b^ |
| Short stature; height centile | No; 91% | No; 81% | No; 14% | No; 27% | Yes; 2% |  | No; 21% | No; 26% | No; 13% | No; >98% | No; >98% |  | 1/5 | >70% | | 10% **[4]** |
| Weight centile | >98% | >98% | >98% | >98% | <2% |  | 59% | 20% | 21% | 80% | >98% |  | 4/5 (>98%) |  | |  |
| BMI (kg/m^2^)^c^ | 44.1 | 34.3 | 35.5 | 24.5 | 18.1 |  | 17.9 | 16.0 | 16.3 | 13.7 | 23.7 |  | $\bar{x}$=31.3 ± 10.1 | 20.3  ± 5.8^d^ | | NA |
| BSA (m^2^)^e^ | 2.80 | 2.02 | 1.90 | 0.89 | 1.45 |  | 1.02 | 0.53 | 1.00 | 0.81 | 1.16 |  | 1.8 |  | |  |
| Body description  (weight centile)^f^ | Obese  (>98%) | Obese  (94%) | Obese  (97%) | Obese  (>98%) | Normal  (11%) |  | Overweight (75%) | Obese  (45%) | Normal  (50%) | Normal  (2%) | Obese  (96%) |  | 4/5 Obese | Obese12.9% **[4]**  >9yr:75-95% **[3]** | | Obese 67% **[4]** |
| **GI symptoms** |  | | | | |  |  | | | | |  |  | | | |
| FTT/feeding issues (infancy) | No | No | Yes | Yes | Yes |  | Yes | Yes | Yes | Yes | No |  | 3/5 | 100% **[5]** | | NA |
| GE reflux (infancy) | No | Yes | No | Yes | No |  | NR | Yes (Mild) | No | Yes | No |  | 2/5 | Increased **[6]** | | NA |
| Constipation | Yes | No | Yes | No | No |  | No | Mild | Yes | Yes (<12mo) | Yes |  | 2/5 | 53% **[7]** | | NA |
| **Craniofacial** |  | | | | |  |  | | | | |  |  | | | |
| Head circumference | >95% | 75% | 50% | 50% | 2% |  | 45% | 75% | 25% | 25% | >98% |  | 3/5 normal | Normal **[3]** | | NA |
| Ocular abnormalities | Left  esotropia | Hyperopia | Strabismus | Strabismus; mild esotropia; dacryostenosis | Mild esotropia |  | No | Pseudo-strabismus, hyperopia | Hyperopia; astigmatism | Myopia | No |  | 4/4 | 50-80% | | 40-60% |
| Dental abnormalities | Yes | No | Missing 2^nd^ premolars | Frequent caries | Caries, overbite; malocclusion |  | NR | Anterior overbite | Dental capping; malocclusion | NR | Yes |  | 4/5 | >90% **[8]** | | NA |
| Cleft lip/palate | No | Bifid uvula | No | No | No |  | No | No | No | No | No |  | 1/5 | 0-10% | | 0% |
| **Skeletal anomalies** |  | | | | |  |  | | | | |  |  | | | |
| Scoliosis | Mild (8y) | Mild (17y) | Kyphoscoliosis | No | No |  | No | No | Thoracolumbar | No | No |  | 3/5 | 40-70% | | 36% |
| Vertebral anomalies  (by x-ray) | Yes, scalloping lumbar, SBO | No | No | No | No |  | No | No | Yes, SBO | No | No |  | 1/5 |  | |  |
| Brachydactyly  (Small hands/feet) | Yes | Yes  Dorsal edema | No | Yes | Yes |  | Yes | No | Yes | No | Yes |  | 4/5 | 80-90% | | 83% |
| **Otolaryngologic/Immune abnormalities** | | | | | |  |  | | | | |  |  | | | |
| Frequent otitis media | Yes | Yes | Yes | Yes | Yes |  | Yes Mastoidectomies | Yes | Yes | Yes (infancy) | Yes |  | 5/5 | 80-90% | | 55% |
| Hearing loss | Left HF-SNHL | Mild-moder. CHL | Right SNHL | Mild CHL | Not tested |  | Left HL | Normal BAER | LFHL | Normal hearing | Normal hearing |  | 4/4 | 60-70% | | 10-25% **[4]** |
| Hoarse, deep voice | Yes | Yes | Yes | Yes | Yes |  | Yes | Non-verbal | No | No | Yes |  | 5/5 | >80% | | 100% |
| Other infections (URIs, sinusitis, pneumonia, etc.) | Yes | No | Yes | Yes | No |  | Yes | No | Yes | Yes | Yes |  | 3/5 | NA | | NA |
| Immunologic abnormalities | No | No | No | No | NR |  | Yes, IVIG | Low IgA | No | High IgG & IgE | High IgE |  | 0/4 | 51% **[9]** | | NA |
| **Neurologic/Cognition** | | | | | |  |  | | | | |  |  | | | |
| Variable ID | DD | Global DD | Global DD | Global DD (GM/FM) | Global DD/ID |  | Global DD | Global DD | Global DD | Global DD | DD |  | 5/5 | 100% | | 100% |
| Recent or last  full scale IQ | Mild ID | Mild ID  (FSIQ 68 at 17y) | Mild ID  (FSIQ 62  at 13y) | Mild ID  (FSIQ 62  at 4y) | Moderate ID |  | Mild ID  (FSIQ 62) | NA | Borderline ID (IQ 76 at 12.5y) | At age 33mo, cognitive was 13mo | Cognitive and speech delay |  | 4/5 mild ID  1/5 moder. ID |  | |  |
| Speech delay | No | No | Yes | Yes | Yes |  | Yes | Yes | Yes | Yes | Yes |  | 3/5 | >90% | | 70% |
| Motor delay | Yes | Yes | Yes | Yes | Yes |  | Yes | Yes | Yes | Yes | No |  | 5/5 | >90% | | 70% |
| Infantile hypotonia | Yes | Yes | Yes | Yes | Yes |  | Yes | Yes | Yes | Yes | No |  | 5/5 | >90% | | 44-61% **[4]** |
| Seizures by history | Yes (tonic clonic) | Yes (febrile; petit mal) | Yes (infancy; motor tics) | No | Yes (<5y) |  | Yes | No | Yes (partial complex) | Yes (multifocal) | No |  | 4/5 | 11-30% **[4]** | | 17% |
| EEG abnormalities | Normal (5y) | Abnormal | Normal | NR | NR |  | Abnormal | Normal | Abnormal | Abnormal | Abnormal |  | 1/3 | 57% **[10]** | | NA |
| Sleep disturbance | Yes | Yes | Yes | Yes | Yes |  | Yes | Yes | Yes | Yes | Yes |  | 5/5 | >90% | | 100% |
| OSA symptoms | Yes | Yes (T&A 2y) | Mild | Yes (T&A 3y) | Yes (T/A 9y) |  | No | Mild | Yes | NR | Yes (T&A 3y) |  | 5/5 | NA | | NA |
| **Neurobehavioral features** | | | | | |  |  | | | | |  |  | | | |
| Attention seeking | Yes | Yes | Yes | Yes | Yes |  | Yes | Not significant | Yes | No | NR |  | 5/5 | 80-100% | | 100% |
| Aggressive outbursts/tantrums | Yes | Yes | Yes | Yes | Yes |  | Yes | Yes | Yes | No | Yes |  | 5/5 |  | |  |
| Anxiety/rapid mood shifts/emotional lability | Yes | Yes | Yes | Yes | Yes |  | Yes | No (28mo) | Yes | NR | No |  | 5/5 | 89% **[11]** | | NA |
| Hugging self/others; hand wringing | Yes | No | No | Yes | Yes |  | Yes | Yes | Yes | Yes | No |  | 3/5 | 50-80% | | 100% |
| Other repetitive /obsessive behaviors | Yes | No | Yes | Yes | Yes |  | Yes | No | Yes | Yes | Yes |  | 4/5 |  | |  |
| Problems regulating food intake | Yes | Yes | Yes | Yes | Yes/Pica |  | No | NR | Yes | NR | Yes/Pica |  | 5/5 | 25% **[4]** | | 86% **[4]** |
| Self-injurious behavior | Yes | Yes | Yes | Yes | Yes |  | Yes | Yes | Yes | No | Nail bites only |  | 5/5 | 75-92% | | 100% |
| Onychotillomania (nail damage/yanking) | Yes | Yes | Yes | Yes | Yes |  | Yes | No | No | No | Yes |  | 5/5 | 25-85% | | 90% |
| Polyembolokoilamania | Yes | Yes | No | Yes | No |  | No | No | Yes | Yes | Yes |  | 3/5 | 25-85% | | 80% |
| **Cardiovascular defects** | Normal ECHO syncope | Normal ECHO | Normal ECHO murmur | Normal ECHO | No |  | Murmur at birth | Normal ECHO | LVH, hypertension; normal ECHO | No | Functional Still's murmur |  | 0/5 structural | <25% | | 0% |
| **Genitourinary anomalies** | | | | | |  |  | | | | |  |  | | | |
| Renal anomalies | No | No | No | No | NR |  | No | No | No | NR | No |  | 0/4 structural | 15-30% | | 0% |
| Frequent UTIs | No | Yes | Yes | 1 at 5y | No |  | No | No | No | No | No |  | 3/5 UTI | <20% **[4]** | | 0% |
| Incontinence/enuresis | No | Enuresis <15y | Enuresis <11y | Enuresis | Enuresis |  | Occasional Incontinence | Enuresis (5y) | Incontinence | Incontinence | Enuresis |  | 4/5 | 82% **[7]** | | NA |
| Genital anomalies | Hypogonado-tropic hypogonadism | No | No | Labial adhesions | No |  | Delayed puberty | No | Premature adrenarche | No | No |  | 2/5 | 6/16 **[12]** | | NA |
| Abbreviations: AGA, appropriate for gestational age; BAER, brainstem auditory evoked response; BMI, body mass index; BSA, body surface area; CHL, conductive hearing loss; DD, developmental delay; ECHO, echocardiogram; EEG, electroencephalogram; EL, expressive language; F, female; FSIQ, full scale IQ; FTT, failure to thrive; GE, gastroesophogeal; GI, gastrointestinal; GM/FM, gross motor/fine motor; HFHL, high frequency hearing loss; HL, hearing loss; ID, intellectual disability; IUGR, intrauterine growth retardation; IVIG, intravenous immunoglobulin; LFHL, low frequency hearing loss; LGA, large for gestational age; LVH, left ventricular hypertrophy; M, male; mo, months; moder., moderate; NA, not available; NR, not reported; OM, otitis media; OSA, obstructive sleep apnea; RL, receptive language; SBO, spina bifida occulta variant; SD, standard deviation; SGA, small for gestational age; SNHL, sensorineural hearing loss; T&A, tonsillectomy/adenoidectomy; URIs, upper respiratory infections; UTI, urinary tract infections; wk, weeks; y, years.  ^a^ M2543 is an ‘*unclassified*’ case  ^b^Average gestation age for published *RAI1* mutation cases (n=8) is 40.7 weeks (AGA) **[13,14,15,16]**  ^c^ Body Mass Index (BMI, kg/m2) calculated at <http://www.halls.md/body-surface-area/bsa.htm>  ^d^ Calculated from the NIH cohort of common 17p11.2 SMS deletion cases (n=49)  ^e^ Body Surface Area (BSA, m2) calculated at <http://www.halls.md/body-surface-area/bsa.htm> with the Mosteller formula  ^f^ Weight centile compared to others of the same height (<http://www.halls.md/body-surface-area/bsa.htm>) | | | | | | | | | | | | | | | | |

**References of Supporting Table S2:**

1. Smith AC, McGavran L, Robinson J, Waldstein G, Macfarlane J, et al. (1986) Interstitial deletion of (17)(p11.2p11.2) in nine patients. Am J Med Genet 24: 393-414.

2. Stratton RF, Dobyns WB, Greenberg F, DeSana JB, Moore C, et al. (1986) Interstitial deletion of (17)(p11.2p11.2): report of six additional patients with a new chromosome deletion syndrome. Am J Med Genet 24: 421-432.

3. Smith ACM, Leonard AK, Gropman A, Krasnewich D. Growth Assessment of Smith-Magenis Syndrome (SMS); 2004. Am Soc Hum Genetics, Toronto, Canada, Oct 2004, Poster 700.

4. Edelman EA, Girirajan S, Finucane B, Patel PI, Lupski JR, et al. (2007) Gender, genotype, and phenotype differences in Smith-Magenis syndrome: a meta-analysis of 105 cases. Clin Genet 71: 540-550.

5. Gropman AL, Duncan WC, Smith AC (2006) Neurologic and developmental features of the Smith-Magenis syndrome (del 17p11.2). Pediatr Neurol 34: 337-350.

6. Smith ACM, Gropman A (2005) Smith-Magenis Syndrome. In: Clinical Management of Genetic Syndromes, 2nd Edition Cassidy S & J Allanson (Eds) Wiley-Liss, New York, NY, 2005.

7. Smith AC, Dykens E, Greenberg F (1998) Sleep disturbance in Smith-Magenis syndrome (del 17 p11.2). Am J Med Genet 81: 186-191.

8. Tomona N, Smith AC, Guadagnini JP, Hart TC (2006) Craniofacial and dental phenotype of Smith-Magenis syndrome. Am J Med Genet A 140: 2556-2561.

9. Introne W, Jurinka A, Krasnwich D, Candotti F, Smith A (2005) Immunologic Abnormalities in Smith-Magenis syndrome (del 17p11.2). Am Soc Hum Genetics, Salt Lake City, Oct, 2005 Poster 605.

10. Potocki L, Shaw CJ, Stankiewicz P, Lupski JR (2003) Variability in clinical phenotype despite common chromosomal deletion in Smith-Magenis syndrome [del(17)(p11.2p11.2)]. Genet Med 5: 430-434.

11. Dykens EM, Smith AC (1998) Distinctiveness and correlates of maladaptive behaviour in children and adolescents with Smith-Magenis syndrome. J Intellect Disabil Res 42 ( Pt 6): 481-489.

12. Stratton RF, Dobyns WB, Greenberg F, DeSana JB, Moore C, et al. (1986) Interstitial deletion of (17)(p11.2p11.2): report of six additional patients with a new chromosome deletion syndrome. Am J Med Genet 24: 421-432.

13. Bi W, Saifi GM, Girirajan S, Shi X, Szomju B, et al. (2006) RAI1 point mutations, CAG repeat variation, and SNP analysis in non-deletion Smith-Magenis syndrome. Am J Med Genet A 140: 2454-2463.

14. Bi W, Saifi GM, Shaw CJ, Walz K, Fonseca P, et al. (2004) Mutations of RAI1, a PHD-containing protein, in nondeletion patients with Smith-Magenis syndrome. Hum Genet 115: 515-524.

15. Girirajan S, Elsas LJ, 2nd, Devriendt K, Elsea SH (2005) RAI1 variations in Smith-Magenis syndrome patients without 17p11.2 deletions. J Med Genet 42: 820-828.

16. Slager RE, Newton TL, Vlangos CN, Finucane B, Elsea SH (2003) Mutations in RAI1 associated with Smith-Magenis syndrome. Nat Genet 33: 466-468.
